# Amidoxime Functionalization of Algal/Polyethyleneimine Beads for the Sorption of Sr(II) from Aqueous Solutions

**DOI:** 10.3390/molecules24213893

**Published:** 2019-10-29

**Authors:** Yuezhou Wei, Khalid A. M. Salih, Siming Lu, Mohammed F. Hamza, Toyohisa Fujita, Thierry Vincent, Eric Guibal

**Affiliations:** 1 Guangxi Key Laboratory of Processing for Non-ferrous Metals and Featured Materials, School of Resources, Environment and Materials, Guangxi University, Nanning 530004, China; yzwei@gxu.edu.cn (Y.W.); Immortaltiger7@gmail.com (K.A.M.S.); lusiming0302@icloud.com (S.L.); fujitatoyohisa@gxu.edu.cn (T.F.); 2 Nuclear Materials Authority, POB 530, El-Maadi, Cairo, Egypt; 3 C2MA, IMT–Mines Ales, Univ. Montpellier, F-30319 Alès cedex, France; Thierry.Vincent@mines-ales.fr

**Keywords:** algal-alginate-polyethyleneimine beads, amidoximation, uptake kinetics, sorption isotherm, desorption, selectivity seawater, strontium

## Abstract

There is a need for developing new sorbents that incorporate renewable resources for the treatment of metal-containing solutions. Algal-polyethyleneimine beads (APEI) (reinforced with alginate) are functionalized by grafting amidoxime groups (AO-APEI). Physicochemical characteristics of the new material are characterized using FTIR, XPS, TGA, SEM, SEM-EDX, and BET. AO-APEI beads are tested for the recovery of Sr(II) from synthetic solutions after pH optimization (≈ pH 6). Uptake kinetics is fast (equilibrium ≈ 60–90 min). Sorption isotherm (fitted by the Langmuir equation) shows remarkable sorption capacity (≈ 189 mg Sr g^−1^). Sr(II) is desorbed using 0.2 M HCl/0.5 M CaCl_2_ solution; sorbent recycling over five cycles shows high stability in terms of sorption/desorption performances. The presence of competitor cations is studied in relation to the pH; the selectivity for Sr(II) is correlated to the softness parameter. Finally, the recovery of Sr(II) is carried out in complex solutions (seawater samples): AO-APEI is remarkably selective over highly concentrated metal cations such as Na(I), K(I), Mg(II), and Ca(II), with weaker selectivity over B(I) and As(V). AO-APEI appears to be a promising material for selective recovery of strontium from complex solutions (including seawater).

## 1. Introduction

Strontium makes up about 0.03% of Earth’s crust, being mainly present in ores as sulfate and carbonate salts (celestine and strontianite mineral, respectively). Natural strontium is not recognized as a very toxic element; however, its chemical properties are very similar to calcium and may cause trouble in the case of excess exposure, especially in the early bone-forming years. This similarity to Ca and Ba is essentially critical because of its competitive assimilation in bones (due to ion-exchange with Ca), especially for ^90^Sr issued from nuclear decay chains. Radioactive strontium ^90^Sr is considered one of the most hazardous radionuclides produced from nuclear fission of ^235^U and ^239^Pu (spent fuel reprocessing and nuclear accidents) [1]. Its half-life time is about 30 years; this is a beta emitter (0.546 MeV). Radioactive ^90^Sr easily accumulates in the human body (including bones) and is excreted difficultly. This intake causes bone sarcoma, leukemia, and soft tissue cancer [2].

The contamination of water bodies with strontium may have different sources: (a) naturogenic (alteration of sedimentary rock formations), (b) anthropogenic (including manufacturing of ceramics, pigments, glasses or electrometallurgy for the production of aluminum for transport industries, or by-product of mining industries), or (c) from the fission industry (^90^Sr from nuclear power units and accidental discharge of contaminated water flow). Essential industrial uses of strontium consist of glace faceplate materials in TV screens and fluorescent lights, ceramic ferrite magnets, ceramic and glass application, pyrotechnic, and paintings. So apart from radioactive strontium in nuclear explosions and as sub-products of radioelement decay, strontium can be found in a large number of effluents from these industrial sectors.

The intake of excessive amounts of strontium may cause serious diseases, especially in bones (cation-exchange between calcium and strontium), with an acute effect on infants and young children. However, the clinical data are rather limited, and Health Canada reports a maximum allowable concentration of 7.0 mg Sr L^−1^ (for natural Sr(II)) [3]. 

Precipitation [4], ion exchange and chelating materials [5,6,7], and membrane techniques [8,9] are the techniques most frequently reported in the literature for metal removal and recovery. Sorption processes involving inorganic exchangers are generally designed for the treatment of effluents containing low concentrations of Sr(II) (especially for radioactive strontium). Indeed, precipitation processes may face difficulties for achieving required levels of removal; and membrane processes are very exigent in terms of energy requirements (in addition, they produce large volumes of concentrates) [10]. Inorganic extractants are “playing” with nanostructured characteristics, ionic cages for capturing radioelements [2,5,11], although porous organic cages (POC) have also been developed for Cs(I) and Sr(II) binding [12]. However, for non-radioactive Sr(II) (as generated by more conventional industries), sorbents bearing specific functional groups are more attractive [13,14]. Many biosorbents have been tested for Sr(II) recovery from dilute solutions, including moss [7], bacteria [15], yeast [16], fishbone [17], algal biomass [18,19], biopolymers [20,21]. Carbon-based sorbents were also used for Sr(II) removal from aqueous solutions [22,23,24,25]. Mineral sorbents, such as clays [6,26,27], silica, and titanate, have retained great attention under their raw form or after impregnation with extractants [28,29], or after immobilization of solid ion-exchangers [30,31].

Algal biomass has been widely studied for the last few decades for the recovery of heavy metal ions using the proper reactivity of the reactive groups present on their constituents directly (i.e., carboxylate groups from alginate, amine groups from proteins, sulfonic groups from fucoidan, etc). Recently, the ability of algal biomass to be conditioned under different forms, such as pure algal beads [32] or algal foams, opened a new perspective for their applications in fixed-bed columns and dynamic systems (e.g., reactive sponges with highly percolating properties) [33,34,35]. The reactivity of algal biomass for metal binding can be improved by the incorporation of other functional groups. For example, by reaction with polyethyleneimine (PEI) and crosslinking, it is possible to synthesize polyfunctional sorbents [32]: the incorporation of PEI can be carried out heterogeneously (incorporation of microparticles of crosslinked PEI) or homogeneously (post-crosslinking of PEI, after intimate mixing and impregnation) [36]. It may be useful adding alginate in the synthesis to improve the resistance of the composite material. The one-pot synthesis is based on the partial extraction of alginate from algal biomass, the ionic interaction between carboxylic groups of alginate and amine groups of PEI, followed by the ionotropic gelation of residual carboxylic groups with calcium and the crosslinking of residual amine groups with glutaraldehyde. This double crosslinking and the interpenetration of polymers produce very stable and highly reactive beads (APEI, algal/PEI beads) for the sorption of heavy transition metal ions. 

The current work describes the functionalization of these composite beads (APEI) with specific functional groups in order to improve the reactivity of the sorbent for strontium. The functionalization is based on the nitrilation of amine groups on the composite, followed by the proper amidoximation of the intermediary product. The first part of the study includes an extensive characterization of the materials to clarify the functionalization mechanism and identify the reactive groups and their modes of interaction with Sr(II). In a second step, the sorption properties are investigated through the study of pH effect, uptake kinetics, sorption isotherms, effects of competition (multi-component solutions), metal desorption, and sorbent recycling. Finally, the sorbent is tested for the removal of Sr(II) from two seawater samples as an example of complex media.

## 2. Results and Discussion

### 2.1. Characterization of Materials

#### 2.1.1. SEM and EDX Characterization

Table S1 (see Supplementary Information, SI) shows the SEM-EDX analysis of the surface of the beads (APEI): (a) before and (b) after chemical modification (i.e., after nitrilation (CN-APEI), (c) after amidoximation (AO-APEI)), (d) after Sr(II) binding, (e) after metal desorption, (f) after five cycles of sorption and desorption, and (g) after exposure to NaCl solution. The surface morphologies of APEI and CN-APEI beads are roughly smooth, with some irregularities and large superficial holes. After functionalization with amidoxime groups (AO-APEI), the surface becomes more irregular with thin wrinkles and sharp edges. After Sr(II) sorption, the surface progressively loses its smooth aspect with the formation of wider wrinkles and small holes. The desorption of Sr(II) does not significantly change the surface aspect of sorbent beads; the surface hardly changes after five cycles of sorption/desorption (compared with the beads after metal desorption). The exposure of raw beads to NaCl solution causes some surface breakage; amidoximated beads are less sensitive to the ionic strengths (high salinity of the solution); however, the wrinkles are more marked (compared with reference AO-APEI beads in low-ionic strength solutions). The semi-quantitative EDX analyses confirm the progressive increase in the nitrogen content of the materials, from APEI to AO-PEI; this is consistent with the proposed mechanism of functionalization. On the other side, the O content substantially increases at the amidoximation step (from CN-APEI to AO-APEI).

#### 2.1.2. Textural Properties

Figure S1 shows the textural analysis of the sorbent with a specific surface area found close to 40 m^2^ g^-1^, a pore volume of 0.21 cm^3^ g^−1^, and a pore size between 168 Å and 218 Å (based on N_2_ desorption and adsorption isotherms, respectively). These values demonstrate that the sorbent has a porous structure, especially considering the mode of drying applied to the material (i.e., air-drying). Indeed, in the case of alginate beads, Rodriguez-Dorado et al. [37] compared the mode of drying of biopolymer beads dried using supercritical CO_2_ conditions (aerogels), freeze-drying (cryogels), and oven-drying (xerogels). The textural properties were affected by external parameters such as the molecular weight and the mode of coagulation; however, the mode of drying drastically influenced the specific surface area, pore volume, and pore size of the beads. Xerogels have porosity under the detection limit of N_2_ adsorption/desorption equipment, while the cryogels showed specific surface areas in the range 0.8–246 m^2^ g^−1^ (depending on experimental conditions). This is lower than the specific surface area obtained with aerogels (271–537 m^2^ g^−1^). Djelad et al. [38] reported similar trends for chitosan-based composite beads. Despite drastic drying conditions, the synthesis procedure incorporating algal biomass, alginate, and PEI allowed maintaining appreciable textural properties. In the case of zeolite functionalization with amidoxime, Puspitasari et al. [39] reported a specific surface area close to 20 m^2^ g^−1^, and a porous volume close to 0.07 cm^3^ g^−1^.

#### 2.1.3. Thermal Degradation—TGA Analysis

The functionalization of APEI beads changes the thermal degradation of the material (Figure S2, due to the presence of new reactive groups grafted on the composite backbone. Several degradation steps can be identified:

(1) 16–190/200 °C—the release of surface-absorbed water and constitutive water (inner water): the profiles are similar for APEI and AO-APEI (weight loss of 11%–15% and maximum DrDTG close to 97 °C) [40,41,42].

(2) 190/200–264/304 °C—cleavage of saccharide rings and ester groups in alginate [43,44], and analogs present in the algal biomass: the profiles remain close for the two materials (total weight loss: 41.17% for APEI and 34.6% for AO-APEI). Two peaks appear on the DrDTG curve for APEI at 218.6 and 300.12 °C, while for AO-APEI, the curve is relatively stable (variations leveled by the strong peak appearing at higher temperatures). This first step was assigned to the devolatilization of brown algae biomass (*Laminaria digitata*) with a strong exothermic peak at 235 °C.

(3a) 304–560 °C: progressive mass loss for APEI associated with the degradation of PEI, and constituents of algal biomass (end of the main devolatilization step and char formation) [45,46,47]. The weight loss at this stage reaches 37.19% (total weight loss: 78.36%). Two small exothermic peaks are observed at 416.1 °C and 539.9 °C. In the thermal decomposition of crosslinked PEI/silica composite, Liu et al. [48] reported a strong exothermic peak at 308 °C due to the decomposition of the organic fraction; the progressive loss of weight till 800 °C was assigned to the combustion of residual organic material and char.

(3b) 264–474 °C: progressive mass loss (about 34.6%) for AO-APEI (cumulative weight loss: 59.2%). By comparison with APEI thermogram, this specific section is probably associated with the thermal degradation of amidoxime moiety. However, Sahiner et al. [49] observed relatively weak changes in the thermograms of PEI and functionalized PEI (including amidoxime derivative). The differences may be attributed to different modes of interaction in the current complex system (algal biomass-alginate-PEI-glutaraldehyde-Ca).

(4) 474–540 °C: strong weight loss (about 21.8%) associated with the thermal degradation of residual organic compounds and combustion of the char. The thermal degradation of AO-APEI requires a higher temperature than APEI. The strong exothermic peak is observed at 484.2 °C.

The total weight losses are very close for the two materials: 78.4% for APEI and 81.0% for AO-APEI. The final solid probably corresponds to the inert calcium-based residue (alginate/algal biomass being ionotropically gelled with CaCl_2_, in addition to the residues of calcium carbonate used for partial alginate extraction during the shaping/conditioning of APEI). The complexity of the structure of algal biomass (due to the presence of different polysaccharides and other cellulose-like or protein constituents) makes the interpretation of the profiles difficult. Anastasakis et al. [50] reported significant changes in the characteristic degradation temperatures of the most representative polysaccharides of brown macroalgae. This complexity is even reinforced by the additional contribution of PEI moieties.

#### 2.1.4. Chemical Characterization of Sorbent—FTIR and XPS Analyses

Figure S3 shows the FTIR spectra of the different materials: not only for successive compounds produced during the functionalization of APEI beads but also after metal sorption, metal desorption, and after five recycling cycles. Table S2 shows the assignments of the main peaks appearing on these spectra. The peak at 2198 cm^−1^ (C≡N stretching vibration) clearly demonstrates the effective nitrilation of the amine groups with chloroacetonitrile. This band disappears after amidoximation: the functionalization is quantitative. Rahman et al. [51] identified this vibration at 2244 cm^−1^ on poly(acrylonitrile) grafted kenaf cellulose. They also observed the disappearance of this band after amidoximation and the appearance of new bands at 1677 and 1647 cm^−1^ (corresponding to C=N stretching and N-H bending vibrations). The FTIR spectrum of CN-APEI also shows a significant shoulder at 1760–1750 cm^−1^, which is usually assigned to carboxylate groups. For AO-APEI, the band is overlapped with the C=N group of amidoxime; this makes the identification of these amidoxime-based vibrations difficult. Actually, the amidoximation is followed by the widening of the C=N band at 1612 cm^−1^, which is associated with the overlapping of carboxylic/carboxylate groups. This was also assigned to the tautomerization effect on the amidoxime group [47]. The sorption of Sr(II) slightly affects the FTIR spectra; the most relevant changes are the weak shifts of the C-N stretching and C-O symmetric stretching vibrations towards higher wavelengths, the shift of C-N stretching (in primary amines) and C-O-C asymmetric stretching towards lower wavelengths and the slight increase in the wavelength of C=N and C=O stretching vibrations. This confirms that the functional groups most involved in Sr(II) sorption are the reactive groups of amidoxime ligand. However, it is important to note that some of these reactive groups are also present in PEI and alginate-like constituents of algal biomass.

**Figure 1:**
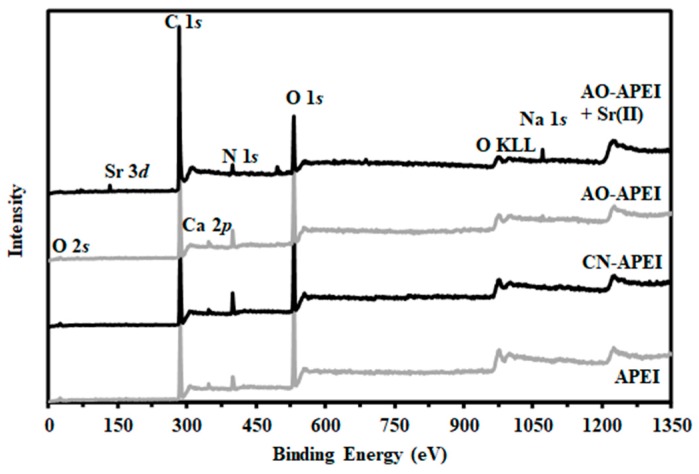
XPS spectra of APEI and derivatives (CN-APEI, AO-APEI) and AO-APEI after Sr(II) sorption (survey spectra).

Figure 1 shows the XPS survey spectra of the materials (at different steps of the synthesis and after metal desorption). The presence of calcium is assigned to the ionotropic gelation of alginate-based materials (for preparing APEI) beads). The N 1*s* peak is identified on the different materials (PEI, proteins of algal biomass, associated with the effects of nitrilation and amidoximation). The sorption of Sr(II) is confirmed by the Sr 3*d* peak; the Na 1*s* peak also appears after metal sorption. Sodium hydroxide was used for pH control; therefore, a residual amount of Na(I) ions may co-exist in the sorbent, due to absorption in the porous network or partial ion-exchange with Ca(II) on alginate. Tables S3 and S4 report the HRES spectra of the most representative signals of characteristic elements. Table S5 summarizes the assignments of these peaks and their variations with sorbent synthesis, metal binding, and after metal desorption. Five peaks (relative to C 1*s*) are identified as C(C, H) and adventitious C at 284.27 eV [44], C-(NH, NH_2_) at 285.41 eV [44], C-O, C=N at 286.08 eV [52], C=O, C-O-C at 287.6 eV [53], and O=C-O, O-C-O at 288.44 eV [52]. The N 1*s* signal on raw material (APEI) is deconvoluted into two peaks: at 399.2 eV and 401.34 eV assigned to C-N, C=NH, –NH, and to N^+^, respectively [47,54]. For O 1*s*, three peaks are identified at 529.29 eV, at 530.7 eV, and at 532.27 eV for O-Ca, C=O, and C-O, and OH, respectively [55,56,57]. Table S5 confirms the grafting of nitrile groups; this is correlated to the appearance (or reinforcement) of the C≡N group on C 1*s* at 287.32 eV, N 1*s* at 398.3 eV signals, and by the significant decrease in the atomic fraction of C-NH or C-NH_2_ groups [58]. After the functionalization of the nitrile groups, their atomic fraction (AF) decreases (becoming negligible) with appearance of the band representative of the –C=NOH groups of amidoxime (285.6 eV for C 1*s* signal and 399.91 eV for N 1*s* signal) and reinforcement of C-NH or C-NH_2_ atomic fractions (with little decrease in BE from 285.4 to 284.9 eV). It is noteworthy that the AF of protonated amine (N^+^) decreases after amidoximation, and that the BE of the protonated nitrogen signal is also shifted at the two steps of the functionalization (from 401.3 eV to 400.5 eV); a new band appears at 532.07 eV (associated with O-N bond) for O 1*s* signal. The sorption of Sr(II) mainly affects C-NH/C-NH_2_ (increase in AF and shift of BEs +0.8 eV) while the C-O/C=N signal almost disappears for C 1*s*. The N 1*s* signals for protonated nitrogen and C=NOH disappear, being replaced with a nitrate-like band and associated with the reinforcement of C-N, C=NH, and –NH signals. Regarding O 1*s* signal, the O-Ca and O-N bands disappear while a nitrate-like peak appears with Sr(II) binding at BE: 405.95 eV (AF < 7%) [59]. These results confirm the contribution of –C=NOH and –NH groups for Sr(II) binding.

#### 2.1.5. Determination of pH_PZC_

Figure S4 reports the pH-drift titration of the AO-APEI sorbent. The pH_PZC_ is close to 6.15. In acidic solutions, the sorbent is protonated: highest proton binding is observed at pH_0_ 3–4 (initial pH), followed by a pH increase of about 1 unit. The equilibrium pH varies, under the optimal conditions, between 4 and 5.5. Under neutral or alkaline conditions, the equilibrium pH decreases by less than 1 unit. At higher pH (i.e., above 9), the pH decrease may reach up to 1.5 units. These properties directly control the ability of the sorbent to bind metal species through chelation/ion-exchange properties and the attraction/repulsion mechanism. At pH below pH_PZC_, amine moieties in either amidoxime groups or PEI are protonated while the hydroxyl moieties are not deprotonated: the global charge of the amidoxime group is cationic. On the other hand, the carboxylic groups on alginate have pK_a_ values close to 3.38 and 3.65 for mannuronic and guluronic acid groups, respectively [60], and those of primary, secondary, and tertiary amine groups in branched PEI are 4.5, 6.7, and 11.6, respectively [61]. Das et al. [62] reported pK_a_ values for amidoxime higher than 11. The overall pH_PZC_ is controlled by the relative influences of these different reactive groups and their respective deprotonations. In the case of amidoximated MCM-41, Xiao et al. [63] reported pH_PZC_ value close to 5.35. Lu et al. [47] reported a pH_PZC_ value close to 6.84 for SiO_2_ /Styrene/DVB functionalized with amidoxime groups. In the case of bis-amidoxime polymers, Piechowicz et al. [64] reported that the protonated-neutral proton transition happens around pH 5.5, while the neutral-anionic step occurs at pH close to 1. Both the environment and support of reactive groups strongly influence the acid-base properties of the sorbent.

#### 2.1.6. Modes of Interaction Sorbent/Sr(II)

**Scheme 1:**
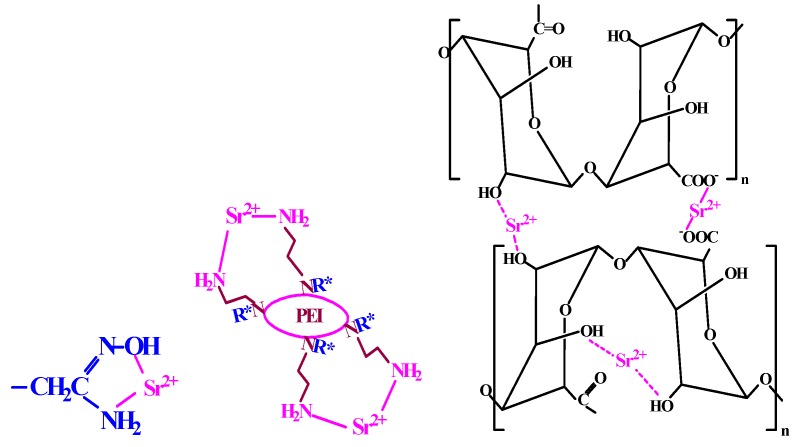
Modes of interaction between Sr(II) and reactive groups on AO-APEI.

Crossing the information collected from FTIR and XPS analyses, the speciation of Sr(II) (only free Sr^2+^ species under selected experimental conditions), and the titration of the sorbent (pH_PZC_), it is possible to suggest different modes of interaction of Sr(II) metal ions with AO-APEI (Scheme 1). In acidic conditions, the protonation of amine and carboxylate groups induces electrostatic repulsion and low sorption capacities (competition of protons against Sr^2+^ for binding on reactive groups). The increase of the pH (above 3.5) leads to the partial deprotonation of carboxylic acid groups (to form carboxylate anions); the progressive deprotonation of amine groups allows the binding of metal cations. On the other hand, free amine groups are essentially protonated in acidic solutions, and with the increase in pH, the repulsion effect decreases: the binding of Sr(II) may occur on carboxylate groups, hydroxyl groups, free amine groups, and amidoxime sites (including –C=NOH and C-NH_2_ groups from the same chain or from vicinal chains). Actually, the co-existence of different reactive groups and their respective influences change with the pH of the solution: protonation/deprotonation, overall charge of the sorbent, and metal speciation. From XPS analysis, Sr(II) may be sorbed by a cation exchange mechanism with Ca(II) bound on carboxylate moieties. Additionally, the high-resolution XPS spectra show that coordinating elements bearing O (hydroxyl groups on saccharide rings, and amidoxime group) as electron-donor groups are also affected by positively-charged Sr(II) binding. Complementary interactions are associated with amine moieties (on amidoxime and on PEI). This diversity of interactions may also explain the relatively high sorption capacities of OA-APEI for Sr(II).

### 2.2. Sorption Properties

#### 2.2.1. The pH Effect

**Figure 2:**
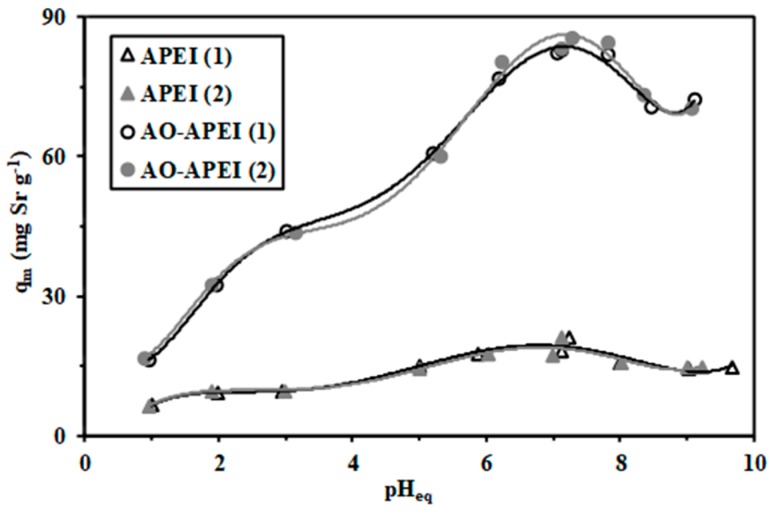
Effect of pH on Sr(II) sorption capacity onto APEI and AO-APEI sorbents—(duplicate experiments; sorbent dosage, SD: 0.375 g L^−1^; C_0_: 29.7 mg Sr L^−1^; temperature: 25 ± 1 °C; agitation speed: 140 rpm; contact time: 48 h).

Figure 2 compares the effect of pH on Sr(II) sorption for APEI and AO-APEI sorbents (including reproducibility testing). The sorption capacity of raw material (APEI) slightly increases with pH, up to 7, and tends to slightly decrease above pH 7.1: the q_eq_ value never exceeds 22 mg Sr g^−1^. The grafting of amidoxime groups significantly improves Sr(II) uptake: the sorption capacity linearly increases with equilibrium pH from 17 mg Sr g^-1^ at pH 1, up to 85 mg Sr g^−1^ at pH 7.3. Above pH 7.8, the sorption capacity tends to decrease but maintains a sorption capacity close to 72 mg Sr g^−1^ at pH 8.3−9.1. Strontium speciation (not shown) in synthetic solutions (prepared with nitrate salts) shows that the metal is present under the form of free Sr^2+^ species, in the whole pH range. So, the pH can only influence strontium sorption through its impact on the sorbent (protonation/deprotonation of reactive groups). In acidic solutions (pH below 2) all the reactive groups held on alginate, algal biomass, PEI, and amidoxime groups are protonated: the protons enter in competition with Sr(II) for metal binding. The sorption capacities remain below 10 mg Sr g^−1^ for APEI. While increasing the pH, the progressive deprotonation of reactive groups increases sorption efficiency: the enhancement is relatively limited for APEI due to a marginal sorption on carboxylate groups, hydroxyls and free amine groups of PEI (not engaged in crosslinking with alginate chains and glutaraldehyde). On the opposite hand, for AO-APEI, the strong increase in sorption capacity means that the amidoxime groups represent the most reactive groups for Sr(II) binding, especially with progressive deprotonation and the decrease of proton competition. This is consistent with the pH_PZC_ value of AO-APEI: at pH below 6.15, the global surface charge is positive, with the highest value around pH 3–4. A progressive decrease is observed between pH 4 and 6. At pH close to 7.3, the sorbent shows maximum Sr(II) sorption capacity. The sorbent is negatively charged (pH being higher than pH_PZC_). Above this value, the sorption begins to decrease because of the possible charge screening effect of Na^+^ (resulting from pH control with NaOH solution) [65,66]. The excess of cations partially shields negatively charged functional groups (such as OH^−^ and COO^−^); in addition, it prevents anion–anion repulsion and increases Na^+^ ion exchange; this was emphasized by the appearance of Na 1*s* signal on the XPS survey analysis of the sorbent after metal sorption. The sorption of Pb(II) on amidoxime-functionalized zeolite hybrids reached an optimum value at pH close to 6 [39]. Rahman et al. [67] reported similar trends for the sorption of Cu(II), Ni(II), Co(II), Cr(III), and Fe(III) on tapioca-cellulose functionalized with poly(amidoxime) ligands. In the case of amidoxime-grafted chitosan, Shaaban et al. [68] observed a continuous increase in sorption capacity between pH 1 and 8 for Sr(II), while for Ba(II) the sorption capacity tended to stabilize at pH 7. For Cu(II) sorption on amidoximated starch embedded in chitosan beads, Dragan et al. [69] reported a strong increase in binding capacity above pH 2 and up to pH 5; it is noteworthy that at pH 1.5, the sorption of the divalent cation was negligible.

Figure S5 shows the pH change during metal sorption. The pH remains stable between pH 1 and 3 and between 7 and 9, while between 4 and 6, the equilibrium pH varies by 1 pH unit. This pH variation does not affect metal speciation and does not cause metal precipitation. The distribution ratio (K_d_ = q_eq_/C_eq_, L g^−1^) is plotted in log_10_ units as a function of equilibrium pH (Figure S6). The plots are characterized by two segments: (a) increasing slope below pH 7.1 (+0.144 and +0.80) (b) decreasing slope above pH 7 (−0.074 and −0.064). The slopes for AO-APEI are higher than for APEI, and the pH has a greater impact on sorption performance for the amidoximated sorbent. For ion-exchange processes, the slope of the curve is usually correlated to the exchange ratio between protons and metal ions. For AO-APEI, this molar ratio is close to 7; this is not consistent with the charge of Sr(II) and its expected interaction with reactive groups (-NH and –NOH). Therefore, other side-reactions are responsible for this high molar ratio.

#### 2.2.2. Uptake Kinetics

**Figure 3:**
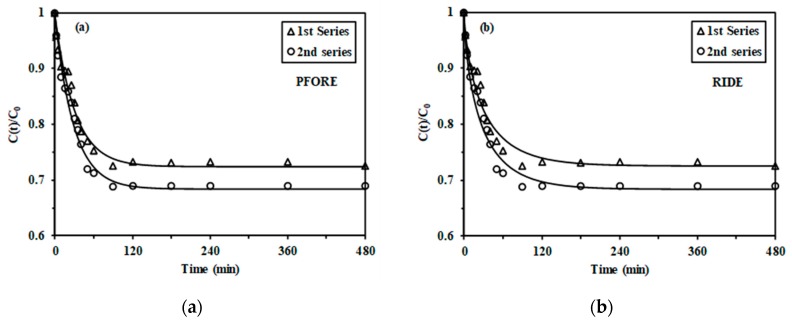
Uptake kinetics for Sr(II) removal using AO-APEI sorbent. Modeling of kinetic profiles with the PFORE (**a**) and the RIDE (**b**) models (pH_0_: 6.0; pH_eq_: 7.1; SD: 0.2 g L^−1^; C_0_: 55.5 and 50.0 mg Sr L^−1^ for 1st and 2nd series, respectively; room temperature: 25 ± 1 °C; agitation speed: 140 rpm).

Uptake kinetics can be controlled by different mechanisms: resistance to diffusion (bulk, film and/or intraparticle, RIDE, resistance to intraparticle diffusion equation) and the proper reaction rate (including the pseudo-first-order rate equation, PFORE, or pseudo-second-order rate equation, PSORE). Figure 3 shows the kinetic profiles (duplicate experiments) for the sorption of Sr(II) using AO-APEI sorbent at pH 6 (equilibrium pH 7.1). The equilibrium is reached within 90–120 min of contact. The kinetic profile is characterized by a first initial section with a steep linear slope: about 50% of total sorption occurs within the first 25 min of contact. The profiles are modeled using the PFORE and the RIDE (solid lines) (the PSORE fit is presented in Figure S7). Table 1 reports the parameters of these models (and the relevant determination coefficients). Best fits were obtained using the PFORE (highest determination coefficients and better approach of equilibrium sorption capacities). The apparent rate coefficient (k_1_) is close to 3.35 × 10^−2^ min^−1^. Dragan et al. [69] reported better fits of kinetic profiles with the PSORE in the case of Cu(II) uptake by amidoxime-based sorbents. In their case, the value of k_1_ was strongly affected by the mode of synthesis; for the most efficient sorbent, the values were comparable to the values found for AO-APEI. Anirhudan et al. [70] also commented that PSORE was more efficient than PFORE for the modeling of kinetic curves for Cu(II) and Zn(II) removal using lignocellulosic support functionalized with amidoxime groups.

Though the resistance to the intraparticle diffusion equation (RIDE, or Crank equation) gives little lower determination coefficients, the kinetic profile is relatively well fitted by the model (Figure 3b). The effective diffusion coefficient is in the range of 6.7–7 × 10^−9^ m^2^ min^−1^. This is about one order of magnitude lower than the value reported for self-diffusion of Sr(II) in water (i.e., 4.75 × 10^−8^ m^2^ min^−1^) [71]. This means that resistance to intraparticle diffusion cannot be neglected in the control of mass transfer of Sr(II) into the sorbent; however, the textural analysis shows that AO-APEI is a porous material (see Section 2.1.2.); this can explain the relatively high diffusion coefficient (only seven times lower than free diffusivity). For amidoxime-fibrous sorbent, Das et al. [62] reported diffusion coefficients close to 4.5 × 10^−8^ m^2^ min^−1^ for U(VI) binding. For uranium recovery from seawater using amidoxime-based polymeric sorbents, Kim et al. [72] found diffusivity coefficients close to 1.05 × 10^−14^ m^2^ min^−1^. The formation of uranium tricarbonate anions hindered the diffusion of metal ions into the polymer compared to uranyl-spiked synthetic solutions (around 6.6 × 10^−9^ m^2^ min^−1^, against 4.67 × 10^−9^ m^2^ min^−1^ for the self-diffusivity of uranyl ions in water [62]). They showed that the limiting kinetic step was the decomplexation of [UO_2_(CO_3_)_3_]^4−^.

#### 2.2.3. Sorption Isotherms

**Figure 4:**
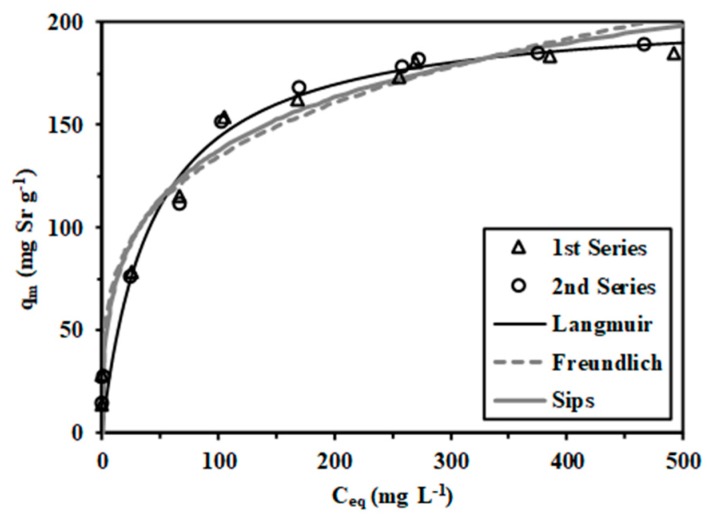
Sorption isotherms for Sr(II) removal using AO-APEI sorbent. Modeling (pH_0_: 6.0; pH_eq_: 7.1; SD: 0.2 g L^−1^; temperature: 25 ± 1 °C; contact time: 48 h; agitation speed: 140 rpm).

Sorption isotherms are reported at pH_0_ 6 (pH_eq_ ranging between 6.8 and 7.3) in Figure 4. The shape of the curve follows the usual Langmuir profile: (a) initial steep increase of sorption capacity followed by (b) a saturation plateau. The maximum sorption capacity tends to 189 mg Sr g^−1^ (2.16 mmol Sr g^−1^). The duplicate experiments confirm the reproducibility in the sorption properties (obtained on two different stocks of beads). 

The experimental profiles are fitted by the Langmuir, Freundlich, and Sips equations. The Freundlich and the Sips equations show very similar fitted curves, which are less accurate than the curve simulated by the Langmuir equation. Table 2 reports the parameters of the models and relevant determination coefficients. The sorption capacity at saturation of the monolayer q_m,L_ is close to 207 mg Sr g^−1^ (i.e., 9.3% higher than experimental value). The affinity coefficient (i.e., b) tends to 2.29 × 10^−2^ L mg^−1^. 

Table 3 compares the sorption properties of a series of sorbents. AO-APEI is part of the most efficient Sr(II) sorbents. The maximum sorption capacity is consistent with the values reported for amidoximated chitosan [72]. However, calixarene-based extractant immobilized on a composite silica/polymer resin shows much higher sorption from nitric acid solutions (i.e., 454 mg Sr g^−1^). The combination of fast uptake and high levels of metal recovery makes the material very attractive.

#### 2.2.4. Multicomponent Sorption

**Figure 5:**
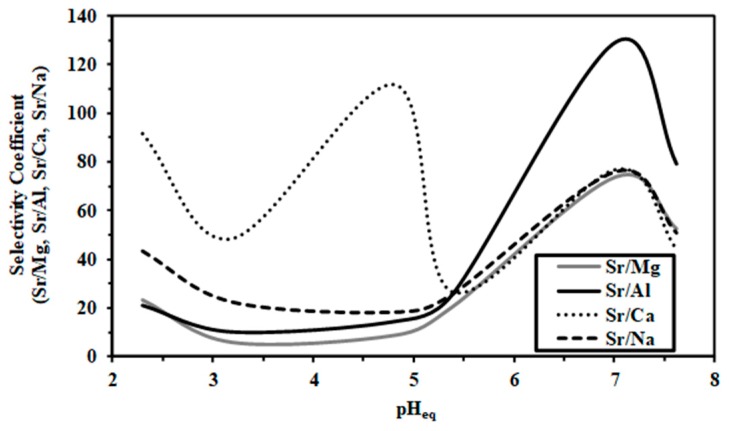
Effect of the pH on the selectivity coefficient for the separation of Sr(II) from Mg(II), Al(III), Ca(II), and Na(I) (equimolar solutions: C_0_ = 1.8 mmol L^−1^; SD: 125 mg L^−1^; contact time: 48 h; agitation speed: 140 rpm: room temperature: 25 ± 1 °C).

The sorption selectivity is an important criterion for designing a separation process. Composite solutions (constituted of equimolar concentrations of different metal ions; i.e., 1.8 mmol metal L^−1^) were treated with AO-APEI, and the residual metal concentrations were analyzed for determining the sorption capacities of the different metals. The selectivity coefficient SC_Sr/metal_ is defined as the ratio of the distribution ratios K_d_(Sr)/K_d_(metal) (with K_d_ = q_eq_/C_eq_, L g^−1^). Figure 5 shows the effect of equilibrium pH on the selectivity coefficients of AO-APEI for Sr(II) over Al(III), Ca(II), Mg(II), and Na(I). The sorbent has a marked preference for Sr(II) over the competitor ions; however, this preference is strongly impacted by the pH and the type of metal. For Al(III), Mg(II), and Na(I), the selectivity profiles follow the same trends: 

(1) At pH 2.3, the SC_Sr/metal_ is higher than 20, 

(2) The SC strongly decreases (down to 8.7–26) when the pH increases (up to 5), 

(3) The SC strongly increases above pH 5 and reaches optimum values as high as SC: 51–129, the maximum selectivity is reported for Sr(II) over Al(III) (i.e., 129 against 76).

(4) Above pH 7, the selectivity tends to decrease again (though maintaining a good SC; i.e., higher than 40). 

In the case of Ca(II), the profiles are roughly the same, the main changes are observed with a maximum SC_Sr/Ca_ close to 111 at pH 4.8 and a relatively high SC (i.e., close to 92) at pH 2.3. 

Table S7 reports the sorption capacities reached at equilibrium and the distribution ratios for the different metal ions at different equilibrium pH values. Figure S8 shows the log_10_-plots of the distribution ratios (K_d_, L g^−1^) for the different metals (in multi-component solutions) as a function of equilibrium pH. Experimental points are linearly distributed with slopes ranging between 0.124 for Al(III) and 0.324 for Ca(II). In order to determine the criteria that control the preference of the sorbent for Sr(II) over the other metal ions, different correlations were tested between these slopes and parameters, such as the Pauling electronegativity, the ionic radius of hydrated metal species [84], or the χ_aq_ electronegativity parameter defined by Li et al. [85]. Actually, the best correlation was obtained for the plot of the slope vs. the softness parameter (Figure S9) [71]. The sensitivity of the selectivity coefficient to pH for the separation of Sr(II) from other selectivity metal cations is controlled by the softness of the metal. Amidoxime (including –N-OH and –NH groups) and amine groups (strong or intermediate hard bases), which are involved in Sr(II) binding, have a general preference for hard acids such as Na(I), Sr(II), Mg(II), Ca(II), and Al(III), according the Pearson’s rules [86]. 

Table S7 reports the SEM observation and semi-quantitative EDX analysis of the surface of AO-APEI at different pH values. Consistently with the mass balance analysis in solution, the increase in pH increases the atomic fraction of metals at the surface of the sorbent. 

#### 2.2.5. Sorption in Fixed-Bed Columns

**Figure 6:**
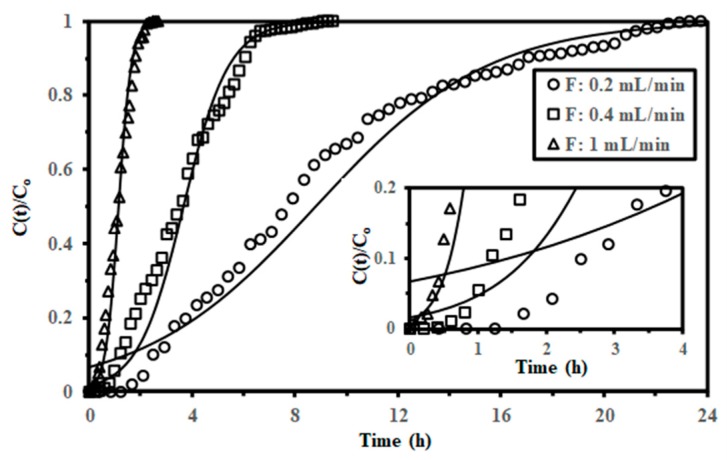
Effect of flow rate on the breakthrough curves for the sorption of Sr(II) using AO-APEI in fixed-bed reactor – Modeling with the Thomas equation [87] (sorbent mass: 70 mg d.w.; bed volume: 5 mL; column depth: 4.4 cm; column diameter: 5 mm; C_0_: 54.3 mg Sr L^−1^; pH_inlet_: 6.0; room temperature: 25 ± 1 °C).

Non-optimal operating conditions have been used for testing the sorption of Sr(II) in fixed-bed columns. Indeed, the ratio of column radius to bead radius was not large enough to effectively minimize the wall effects. However, these preliminary results give the first indication on the possibility of using AO-APEI in a dynamic system. Figure 6 compares the breakthrough curves obtained at different flow rates (for C_0_: 54 mg Sr L^−1^). 

The modeling of experimental breakthrough curves (points) with the Thomas model is represented by the solid curves. The fit of experimental data is improved by high flow rates. Table S8 reports the parameters of the model for the three curves and compares calculated and experimental values for the sorption capacities: they are remarkably close. As the flow rate increases, the sorption capacity tends to decrease: the lower residence time and the wall effects may explain that the sorbent is not fully saturated. The sorption capacity, even at low flow rate, is significantly lower than the value deduced from the Langmuir equation: for C_eq_ = 54 mg Sr L^−1^, the sorption capacity in the batch reactor is found close to 114 mg Sr g^−1^ instead of 80.8 mg Sr g^−1^ in the fixed-bed column. The calculated values for saturation capacity (i.e., q_0_, mg Sr g^−1^) and rate parameter (i.e., k_Th_, L mg^−1^ h^−1^) are correlated with the flow rate (i.e., Q, L h^−1^) according to Equations (1) and (2):

q_0_ = 52.0 Q^−0.271^      (R^2^: 0.999) (1)

k_TH_ = 1.454 × Q − 0.0128      (R^2^: 0.999) (2)

#### 2.2.6. Metal Desorption and Sorbent Recycling

Strontium desorption was tested using a 0.2 M HCl solution. This eluent was enriched with CaCl_2_ (0.5 M) in order to facilitate the ion-exchange mechanism and reinforce the stability of the sorbent; calcium ions contribute to the ionotropic gelation of alginate (carboxylic groups). Figure S10 shows the kinetic profile: the complete desorption of Sr(II) is achieved within 150 min. The desorption step is a little faster than the sorption process. The pseudo-first-order rate and pseudo-second-order rate equations were used for fitting the desorption profile. While the PSORE fits well the initial section of the curve (0–15 min time range), the second section of the curve (15–50 min) obeys the PFORE. Table S9 reports the relevant parameters. The determination coefficients are of the same order (0.983–0.986). Actually, the overall desorption kinetics obeys a variable order rate equation.

This eluent was used for testing the recycling of the sorbent. Table 4 summarizes the efficiencies of sorption and desorption for five cycles (under similar experimental conditions). The performances remain remarkably stable: the sorption efficiency decreases from 60.7% to 56.3% at the fifth cycle. The decrease in desorption efficiency does not exceed 5% (from 96.5% to 91.7%). This good stability is consistent with the characterization of the materials (using FTIR, SEM, and XPS); though some (weak) changes were observed, the physical structure and chemical characteristics were globally maintained (Figure S3, Tables S1 and S2). 

### 2.3. Strontium Removal from Seawater

The Fukushima Daiichi nuclear disaster illustrates a situation where Sr(II) recovery is a critical challenge due to both the presence of radioelements and the elevated ionic strength of the “solution”. It appeared important testing Sr(II) recovery from such a complex medium. Strontium is present in seawater at average concentration close to 7–8 mg Sr L^−1^ [88]; as SrSO_4_ (60%) and free Sr(II) (40%) [89]. The presence of organic ligands strongly affects the speciation of metal ions [90], which, in turn, influences their affinity for the sorbent. Two samples of seawater were collected in China (Beihai) and Vietnam (Da Nan) for investigating Sr(II) recovery from high ionic strength solutions. Figure S11 shows the XPS survey spectra of the sorbent exposed to seawater samples: the binding of Sr(II) can be confirmed by the appearance of the Sr 3*d* signal. Other peaks appear, and they are assigned to U(VI) (U 4*f* signal), B(III) (B 1*s* signal), or Rb(I) (Rb 3*d* signal). In addition to Sr(II), the preliminary semi-quantitative EDX analysis of AO-APEI, after being exposed to seawater, shows the enrichment of the sorbent with metal ions present in large excess (for example: Na(I) and K(I), Mg(II), and Ca(II)) or at trace levels (such as Sr(II), B(III), or As(V)). Figure S12 compares the time-evolution of the selectivity coefficient of Sr(II) against the other metals. It is remarkable that the selectivity reaches a maximum after 18 h of contact before strongly decreasing. As mentioned above, for the discussion of the decrease of the capacity in alkaline solutions, and from the data of pHpzc, the sorbent at this pH is fully negatively charged. The presence of other elements with high concentrations of Al, Mg, Na, and Ca that have a smaller ionic radius (118, 145, 190, and 194 pm, respectively) than Sr (219 pm), may cause a charge screening effect, which, in turn, influences equilibrium performance. In addition, these interactions may cause steric hindrance that affects the swelling properties of the polymer composite network. This effect may influence the mass transfer of Sr(II) through small pores. This kinetic effect is complementary to the equilibrium effect. AO-APEI has a high selectivity for Sr(II) over Na(I), K(I), Mg(II), and Ca(II), and a little preference over B(III) and As(V). 

Table S10 summarizes the main results including: (a) the comparison of initial and residual concentrations, (b) the evaluation of sorption capacities, and (c) the calculation of distribution coefficients after 18 h of contact. The large excess of Na(I), K(I), Mg(II), and Ca(II) cations may explain their high sorption capacities; however, the concentration abatements were negligible and poorly representative; as a consequence, the distribution ratios are also negligible. In the case of trace elements, it is remarkable that Sr(II), B(I), and As(V) show high K_d_ values: around 17, 4.6, and 6–10 L g^−1^, respectively. Figure S13 shows the EDX cartographies for the distribution of metal ions (both at the surface and in the cross-section of the sorbent): Sr(II) and U(VI) (as examples) are homogeneously distributed on AO-APEI. Table S11 reports the semi-quantitative EDX analysis of the sorbent (surface and interior) after being exposed to seawater samples. This table allows identifying the affinity of AO-APEI for several metals and the significant accumulation of elements such as B, U, Sr, and Cs: compared to their low concentrations in seawater, their enrichments on the sorbent are remarkable. It is also noticeable that the distribution is globally homogeneous for U, B, Sr, while Cs tends to accumulate preferentially at the surface of the material.

### 2.4. Noticeable effect of the Amidoximation Process on the Stability of the Sorbent in Complex Environment

The exposure of APEI beads to high ionic strength solutions (such as 2 M NaCl solution for 48 h) leads to a progressive loss of physicochemical stability: the ion-exchange between Na(I) and Ca(II) destabilizes the ionotropic gelation of the beads and the GA-PEI crosslinking is not sufficient for maintaining the integrity of the sorbent. On the opposite hand, amidoximation functionalization (which includes complementary crosslinking) reinforces the stability of the material. It was not possible to detect a change in mechanical fragility. XPS analysis on APEI and AO-APEI before and after exposure to 2 M NaCl solutions for 48 h gives complementary evidence of the improvement of the chemical stability of the material after amidoximation (Tables S12–S14). Some peaks (more specifically associated with ester and O-C-O bonds) disappear on APEI. In the case of AO-APEI, the spectra are poorly affected by the long contact-time with NaCl solutions, and most of the changes are assigned to the little shifts of some BEs.

## 3. Materials and Methods

### 3.1. Materials

Brown algae (*Laminaria digitata*) was provided by Setalg (Pleubian, France). After washing, drying, and grinding, the biomass was sieved to recover the fraction below 250 µm. Alginate (Manugel GMB) was supplied by DuPont (Landerneau, France; now JRS Rettenmaier). Branched polyethyleneimine (PEI, 50% (*w*/*w*) in water) and glutaraldehyde (GA, 50% (*w*/*w*) in water) were purchased from Sigma-Aldrich (Taufkirchen, Germany) Sodium carbonate, and calcium chloride were obtained from Chem-Lab NV (Zedelgem, Belgium). Strontium nitrate (Sr(NO_3_)_2_) was used for lab experiments (Sr(NO_3_)_2_) (single metal solutions) while strontium chloride (SrCl_2_) was selected for multi-component solution; both of them were purchased from the Damao Chemical Reagent Factory (Tianjin, China) and Sinopharm Chemical Reagent Co., Ltd. (Shanghai, China), respectively. MgCl_2_.6H_2_O, AlCl_3_.6H_2_O, CaCl_2_, NaCl, Anhydrous K_2_CO_3_ were supplied by Guangdong Guanghua Sci-Tech Co., Ltd. (Guangdong, China). Ethylene glycol diglycidyl ether, Chloroacetonitrile, and hydroxylamine hydrochloride were purchased from Shanghai Makclin Biochemical Co., Ltd. (Shanghai, China).

The process was developed using *Laminaria digitata*, an emblematic example of abundant brown algae, that was already investigated for metal sorption and widely characterized. The process could be extended to other brown algae containing substantial amounts of alginate (i.e., higher than 25%–30%, *w*/*w*).

### 3.2. Synthesis of Algal/PEI Beads

The algal/PEI beads were prepared according a method previously described [36]. Briefly, 18.75 g of dry algal biomass was dispersed into 750 mL of Na_2_CO_3_ solution (1%, *w*/*w*) under agitation and heating (50 °C) for 24 h. After cooling, a volume (i.e., 250 mL) of alginate solution (4%, *w*/*w*) was added to biomass suspension. Five milliliters of PEI solution (50%, *w*/*w*) were then added to 500 mL of the mixture under agitation. The algal biomass/alginate/ PEI suspension was distributed through a thin nozzle into a 1-L volume of a solution containing both CaCl_2_ (1%, *w*/*w*) and GA (5 mL, 50%, *w*/*w*). The beads (APEI) were maintained under agitation overnight in the crosslinking solution before being filtrated, rinsed with tap water, and freeze-dried.

### 3.3. Functionalization of Algal/PEI Beads (APEI Beads)

The different steps of the functionalization process are described by Scheme S1.

#### 3.3.1. Crosslinking of APEI Beads

In order to reinforce the chemical stability of the beads (especially under the experimental conditions selected for next functionalization steps), they were crosslinked using poly(ethyleneglycol) diglycidyl ether (3 mL) diluted into isopropyl alcohol (90 mL). The suspension was maintained in agitation under reflux for 4 h. After washing with isopropyl alcohol and water, the beads (^C^APEI) were air-dried at 50 °C under vacuum.

#### 3.3.2. Nitrilation of ^C^APEI Beads

Crosslinked beads (^C^APEI, 5 g) were suspended into 150 mL of dried dimethylformamide (DMF) and mixed with 23 g of anhydrous potassium carbonate, under gentle agitation and reflux (at 70 °C) for 30 min. After cooling, 13.3 mL of chloroacetonitrile were introduced in the reactor, and the mixture was maintained under gentle agitation and reflux for another 4 h. The beads were removed by filtration, washed up with hot water, and rinsed with methanol (several times) to remove unreacted reagents. Finally, the beads (CN-APEI) were dried overnight at 50 °C under vacuum.

#### 3.3.3. Amidoximation of Nitrilated APEI Beads

Twenty-three grams of hydroxylamine hydrochloride were dissolved in 75 mL of methanol/water solution (5:1); the pH of the solution was controlled to 9, using concentrated NaOH solution, to precipitate NaCl [40]. Filtrated solution was used for the amidoximation of the nitrilated beads (CN-APEI) under reflux (at 70 °C) for 5 h [91,92]. The sorbent (AO-APEI) was recovered by filtration, rinsed with water, and air-dried at 50 °C under vacuum.

The macroscopic morphology of APEI and AO-APEI beads (and their sizes) are reported in Table S15.

### 3.4. Characterization of Materials

SEM (scanning electron microscopy) observations were performed on a Phenom ProX SEM (Thermo Fisher Scientific, Eindhoven, The Netherlands) at an accelerating voltage of 15 kV. The chemical composition of the samples was characterized by energy dispersive X-ray analysis (integrated to Phenom ProX SEM). 

BET surface area and porosity of the sorbent were recorded by Micromeritics TriStar II (Norcross, GA, USA) system at 77 K and using the BET equation with N_2_ gas and desorption branches of isotherms based on BJH methods, respectively. BET samples were dried for 4 h at 120 °C with N_2_ gas before testing (final degassing was performed again at 120 °C under vacuum). 

The thermogravimetric analysis of materials was performed using TG-DTA equipment (Netzsch STA 449 F3 Jupiter, NETZSCH-Gerätebau HGmbh, Selb, Germany); analysis was carried out under N_2_ atmosphere conditions with a temperature ramp of 10 °C min^−1^ (and a 1 min step at 100 °C).

FT-IR spectrometry analysis was recorded using an IRTracer-100 FT-IR spectrometer (Shimadzu, Tokyo, Japan). All the samples were dried at 60 °C before being analyzed. Samples were conditioned as a KBr disk containing 1% (*w*/*w*) finely ground material particles.

XPS spectra were recorded using an ESCALAB 250XI^+^ instrument (Thermo Fischer Scientific, Inc., Waltham, MA, USA) with monochromatic X-ray Al K_α_ radiation (1486.6 eV) and the following operating conditions: the spot size was 500 μm; the sample-preparation pressure was set to 10^-8^ mbar. The calibration of the energy interval was set on Ag 3*d_5/2_* signal (ΔBE: 0.45 eV) and C 1s signal (ΔBE: 0.82 eV). The full-spectrum pass energy and narrow-spectrum pass energy were set at 50 eV and 20 eV, respectively.

The pH-drift method was used for the determination of the pH of zero charge (pH_ZPC_) [42]. A series of solutions were prepared with the initial pH (pH_0_) varying between 1 and 11 using NaCl as the background salt (at the concentration of 0.1 M). The material was shaken for 48 h with the different solutions with a sorbent dosage of 2 g L^−1^. The sorbent was filtered from the solution, and the equilibrium pH was measured using an S220 Seven Compact pH/ Ionometer (Mettler-Toledo Instruments, Shanghai, China). The equilibrium pH was measured and compared to the initial pH value. The pH_PZC_ was defined by the pH value verifying pH_0_ = pH_eq_.

### 3.5. Sorption Procedures

Most of the experiments were performed in batch tests. A fixed volume (L) of solution (at concentration C_0_, mmol Sr L^−1^) and fixed pH value was mixed with a given amount of sorbent (m, g) for variable contact times (uptake kinetics) or at equilibrium time (set to 48 h). Collected samples were filtrated on filter membranes (1.2 µm) and analyzed for residual metal concentration (C_eq_, mmol Sr g^−1^) using an inductively coupled plasma atomic emission spectrometer (ICPS-7510 Shimadzu, Tokyo, Japan). The sorption capacity at equilibrium (q_eq_, mmol Sr g^−1^) was calculated using the mass balance equation: q_eq_ = (C_0_ − C_eq_)V/m. For fixed-bed column experiments, the column (5 mm diameter) was filled (depth: 4.4 cm) with a small amount of sorbent (70 mg, d.w.); it is noteworthy that the size of the beads was too large to avoid wall effects, but these preliminary tests give the first indication on the feasibility of the application of these beads for dynamic treatment. The preferential channeling and the loss of packing density close to the wall of the column induce the heterogeneous distribution of the metal ions in the cross-section of the reactor and, consequently, a less effective mass transfer, which leads to non-homogenous transfer of the solution through the bed. To prevent this effect, it is usually necessary to maintain the size ratio bead/column diameters (d/D) less than 1/10 or 1/12. The amount of sorbent available for these dynamic tests (performed at the end of the study) was not sufficient for performing experiments consistent with the appropriate d/D ratio. All experiments were performed at room temperature (i.e., 25 ± 1 °C). Sorption tests in multi-component solutions were carried out to evaluate the selectivity of metal sorption for Sr(II) at different pH values. The solutions containing 1.8 mmol L^−1^ of Mg(II), Al(III), Ca(II), Na(I), and Sr(II) (as chloride salts) at initial pH ranging between 2 and 7 were mixed with the sorbent (sorbent dosage, SD: 0.125 g L^−1^) for 48 h. The mass balance equation allowed calculating the sorption capacity for each metal, to deduce the distribution ratio (K_d_, L g^−1^), and then the selectivity coefficient (SC, dimensionless) of Sr(II) over competitor metal ions: SC = K_d_(Sr)/K_d_(metal).

Desorption tests were carried out in batch system by contact of Sr(II)-loaded beads with eluent solution (0.5 M CaCl_2_/0.2 M HCl) for 2 h, under agitation (150 rpm). The sorbent dosage (SD, g L^−1^) was fixed to 2 g L^−1^. Recycling tests (5 cycles) were carried out using the same procedures for both sorption (S) and desorption (D); a washing step was operated between each S and D operations using 10 mL of demineralized water (twice).

Experiments were tested on complex matrices (seawater) for testing the recovery of Sr(II). The samples were collected on two sites: Beihai (China) and Da Nang (Vietnam); initial pH values were: 7.63 and 7.98, respectively. The sorption tests were carried out in a batch with a sorbent dosage of 0.2 g L^−1^ (at natural pH). Samples were collected at different contact times (up to 96 h) and analyzed by ICP-AES or AAS (AA-7000, Shimadzu, Tokyo, Japan).

Detailed experimental conditions are systematically reported in the caption of figures. The modeling of uptake kinetics and sorption isotherms are summarized in Tables S16 and S17.

## 4. Conclusions

The FTIR and XPS analyses confirm the different steps in the chemical modification of algal/alginate/PEI beads (i.e., nitrilation followed by amidoximation). These techniques also bring information on (a) the modes of interaction involved in Sr(II) sorption (contribution of carboxylic, amine and amidoxime groups), and (b) the stability of the sorbent (AO-APEI) after desorption and recycling. The porous structure (characterized by specific surface area close to 40 m^2^ g^−1^ and pore volume close to 0.21 cm^3^ g^−1^) may explain the relatively fast sorption of Sr(II): equilibrium is reached within 60–90 min. The pseudo-first-order rate equation fits the kinetic profile well; though the resistance to intraparticle diffusion also slightly affects mass transfer, the diffusion coefficient is about one order of magnitude lower than the self-diffusivity of Sr(II) in water. The amidoximation strongly increases sorption capacity (up to 188 mg Sr g^−1^, 2.15 mmol Sr g^−1^). The sorption isotherm is well fitted by the Langmuir equation. Metal desorption is highly efficient using 0.2 HCl/0.5 M CaCl_2_ solutions, and the sorbent can be reused for five cycles of sorption and desorption with a limited decrease in performances. The beads were successfully applied in a dynamic system (fixed-bed columns) though the wall effects (driven by the inadequacy of bead size compared to column diameter) affected binding performance. The Thomas model gives a relatively correct fit of experimental profiles. In multi-metal solutions (including equimolar concentrations of Ca(II), Sr(II), Na(I) Mg(II), and Al(III)), AO-APEI has a marked preference for Sr(II), especially at pH close to 7: the effect of pH (slope of the distribution coefficient) can be correlated to the softness parameter of the metal ions. This preferential affinity for Sr(II) is confirmed by the application to the recovery of Sr(II) from seawater. Despite the presence of a large excess of alkali (such as Na(I) and K(I)) and alkali-earth (such as Ca(II) and Mg(II)) metal ions, the selectivity coefficients for Sr(II) over these metals vary between 200 and 800. This is opening the way for future applications on ^90^Sr recovery from contaminated seawater.

The promising results obtained with amidoxime-functionalized APEI material opens the application for the recovery of strontium from complex solutions. The comparison of sorption properties with more conventional sorbents clearly demonstrates the interest for preparing new resins (APEI) based on renewables resources (algal biomass, alginate) and environmentally friendly polymers (PEI) as precursors. This new generation of APEI beads can serve as supports for manufacturing different functionalized resins; we recently developed a quaternarized functionalized material (Q-APEI) that develops very attractive performances for Sc(III) sorption [93].

The sorbent could be used for Sr removal from radioactive effluents, providing complementary tests show the stability of these materials under irradiation. The issue of metal desorption would be secondary in this case, the objective being concentrating and confining dispersed highly hazardous compounds (at micro-level concentrations).

## Figures and Tables

**Table 1:** Uptake kinetics for Sr(II) sorption onto AO-APEI. The parameters of the models.

Model	Parameter	1st Series	2nd Series
Experiment	q_eq,exp._ (mg Sr g^−1^)	74.6	77.7
PFORE	q_eq,1_ (mg Sr g^−1^)	76.4	79.2
	k_1_ × 10^2^ (min^−1^)	3.27	3.44
	R^2^	0.973	0.981
PSORE	q_eq,2_ (mg Sr g^−1^)	89.2	91.8
	k_2_ × 10^4^ (L mg^−1^ min^−1^)	4.30	4.53
	R^2^	0.957	0.969
RIDE	D_e_ × 10^9^ (m^2^ min^−1^)	6.7	7.0
	R^2^	0.962	0.975

**Table 2:** Sorption isotherms for Sr(II) binding onto AO-APEI. The parameters of the models.

Model	R^2^	Parameter	AO-APEI sorbent
Experiment		q_m,exp._ (mg Sr g^−1^)	189.3
Langmuir	0.988	q_m,L_ (mg Sr g^−1^)	206.9
		b_L_ × 10^2^ (L mg^−1^)	2.29
Freundlich	0.971	K_F_	41.2
		n_F_	3.89
Sips	0.977	q_m,S_ (mg Sr g^−1^)	384.1
		b_S_ × 10^2^ (L mg^−1^)	8.81
		n_S_	2.48

**Table 3:** Comparison of Sr(II) sorption properties for various sorbents.

Sorbent	pH_0_	Time (min)	q_m_ (mg Sr g^−1^)	b×10^2^ (L mg^−1^)	Reference
Clay	3.4 *	300	2.98	0.336	[26]
Bentonite	6	120	38.6	−	[27]
Zeolite microsphere	6	20	109.89	15.72	[73]
Activated porous calcium silicate	7	120	142.3	-	[74]
Oxidized Multiwall CNTs	7	100	6.62	0.608	[22]
Moss	6	240	13.1	5.71	[7]
Zr-Sb oxide/PAN	4.74	270	43.67	84.0	[75]
Resorcinol-formaldehyde-iminodiacetic acid resin	9	1200	188.4	-	[76]
Impregnated resin	1 M HNO_3_	120	4.83	5.8	[77]
Extractant impregnated silica/polymer resin	2 M HNO_3_	360	454.5	0.013	[78]
Ni-K Ferrocyanide/TiO_2_	7.8	180	143	0.61	[31]
Prussian blue analog on chitosan/CNT	6	240	205.1	0.46	[79]
Amidoxime chitosan beads	8.5	180	153	-	[68]
Aminophosphonic chitosan/Co(II)	6	60	3.41	89.0	[80]
*Saccharomyces cerevisiae*	3–4 **	1800	37.4	3.51	[16]
Bacteria-derived carbonaceous nanofibers	4.5	1440	67.11	44.7	[53]
EDTA-mercerized bacterial cellulose membrane	6.0	600	44.86	8.5	[81]
Magnetic chitosan beads	8.2	360	11.58	-	[82]
Alginate beads	6	1440	111.1	9.4	[83]
AO-APEI	6.0	90	189	2.29	This work
					

*: equilibrium pH, **: complex solution (simulate of high-level liquid waste).

**Table 4:** Metal desorption and sorbent recycling. Comparison of sorption and desorption performances for five successive cycles.

	Sorption Efficiency (%)		Desorption Efficiency (%)	
Cycle #	Aver.	St. Dev.	Aver.	St. Dev.
1	60.70	0.14	96.49	0.79
2	60.10	0.76	99.16	1.27
3	59.05	0.56	95.09	2.29
4	57.66	0.27	91.85	4.49
5	56.29	0.26	91.66	1.16

## Supplementary Materials

The following are available online, Scheme S1. Schematic presentation of the different steps involved in APEI functionalization. Table S1. SEM and EDX analysis of the raw material (APEI), nitrilated material (CN-APEI), and amidoxime-functionalized material (AO-APEI), before and after Sr(II) sorption, after Sr(II) desorption and after the fifth cycle of sorption and desorption, and after treatment with sodium chloride for either raw materials and AO sorbent. Table S2. Assignments and characteristic wavenumbers (cm^−1^) of the different peaks of the spectra of sorbent (at the different steps of the synthesis and after metal sorption and after 5 recycling cycles). Table S3. XPS characterization of APEI and CN-APEI materials. Table S4. XPS characterization of AO-APEI before and after Sr(II) sorption. Table S5. Assignments, Binding energies (BEs), and Atomic Fractions (AF, %) on the different stages of synthesis and after sorption. Table S6. Sorption capacities (q_eq_, µmol g^−1^) and distribution ratio (K_d_, L kg^−1^) for metal sorption from multi-metal equimolar concentrations at different equilibrium pH values. Table S7. SEM and EDX analysis of AO-APEI sorbent after loading with multi-metal solutions (Ca(II), Na(I), Mg(II), Al(III), and Sr(II)) at different pH values (i.e., 2–7). Table S8. Modeling of breakthrough curves for the sorption of Sr(II) using AO-PEI—Parameters of the Thomas model. Table S9. Modeling of kinetic profiles for Sr(II) desorption from metal-loaded AO-PEI—PFORE and PSORE equations. Table S10. Summary of the results obtained in the treatment of two seawater samples (after 18 h of contact). Table S11. Semi-quantitative EDX analysis of sorbent (surface and interior; average of 4 or 5 analyses (weight percentage) with standard deviation into parentheses) after treatment of two seawater samples (Beihai, China and Da Nan, Vietnam). Table S12. XPS characterization of APEI before and after exposure to 2 M NaCl solution for 48 h. Table S13. XPS characterization of AO-APEI before and after exposure to 2 M NaCl solution for 48 h. Table S14. Assignments, Binding energies (BEs), and Atomic Fractions (AF, %) of APEI and AO-APEI before and after exposure to 2 M NaCl solution for 48 h. Table S15. Macroscopic morphology of APEI and AO-APEI beads and size measurements. Table S16. Uptake kinetics models—PFORE (pseudo-first order rate equation), PSORE (pseudo-second order rate equation) and RIDE (resistance to intraparticle diffusion equation—Crank equation). Table S17. Sorption isotherm models Figure S1. Textural analysis of AO-APEI sorbent. Figure S2. Thermal analysis (TGA and DrDTG) of APEI and AO-APEI materials (Temperature ramp: 10 °C/min; N_2_ atmosphere). Figure S3. FTIR spectra of APEI, CN-APEI, AO-APEI materials, sorbent after Sr(II) sorption, after metal desorption and after 5 cycles of sorption and desorption (test of sorbent stability at recycling). Figure S4. Determination of the pH_PZC_ of AO-APEI—pH-drift method (Sorbent dosage, SD: 2 g L^−1^; Time: 48 h; Background salt: 0.1 M NaCl). Figure S5. pH variation during Sr(II) sorption using APEI and AO-APEI sorbents—(Duplicate experiments; Sorbent dosage, SD: 0.375 g L^−1^; C_0_: 29.7 mg Sr L^−1^; room temperature: 25 ± 1 °C; agitation speed: 140 rpm; contact time: 48 h). Figure S6. Effect of pH on the distribution coefficient (log_10_ units) for Sr(II) onto APEI and AO-APEI sorbents—(Duplicate experiments; Sorbent dosage, SD: 0.375 g L^−1^; C_0_: 29.7 mg Sr L^−1^; room temperature: 25 ± 1 °C; agitation speed: 140 rpm; contact time: 48 h). Figure S7. Uptake kinetics for Sr(II) removal using AO-APEI sorbent—Modeling of kinetic profiles with the PSORE model (pH_0_: 6.0; pH_eq_: 7.1; Sorbent dosage, SD: 0.2 g L^−1^; C_0_: 55.5 and 50.0 mg Sr L^−1^ for 1st and 2nd series, respectively; Temperature: 25 ± 01 °C; agitation speed: 140 rpm). Figure S8. Effect of pH on distribution coefficients (log_10_ units) for Sr(II), Al(III), Ca(II) Mg(II) and Na(I) recovery using AO-APEI from equimolar multi-component solutions (1.8 mmol L^-1^; SD: 125 mg L^-1^; Temperature: 25 ± 1 °C; agitation speed: 140 rpm; contact time: 48 h). Figure S9. Correlation between the slope of the plots log_10_ K*d* vs. pH and the softness parameter of selected metal ions (data collected from Figure S8 and Marcus). Figure S10. Desorption kinetics for Sr(II) elution from AO-APEI sorbent using 0.5 M CaCl_2_/0.2 M HCl solution—Modeling of kinetic profiles using the PFORE and PSORE adapted to desorption (SD: 1 g L^−1^; q_0_: 76 mg Sr g^−1^ = 0.867 mmol Sr g^−1^; Temperature: 21 ± 1 °C; agitation speed: 140 rpm). Figure S11. XPS (survey) spectra of AO-APEI beads after being in contact with seawater samples (Beihai, China and Da Nan, Vietnam). Figure S12. Selectivity for Sr(II) removal from 2 samples of seawater (SD: 0.2 g L^−1^; pH_0_: 7.98 (Da Nan seawater sample), 7.63 (Biehei seawater sample). Figure S13. Example of EDX analysis (element cartography) of AO-APEI beads after contact with seawater (analysis of surface and section).

## Abbreviations

PEI	polyethyleneimine
APEI	Algal-polyethyleneimine
^c^APEI	Crosslinked Algal-polyethyleneimine with poly(ethyleneglycol) diglycidyl ether
AO-APEI	Amidoxime- Algal-polyethyleneimine
ICP-AES	Inductively coupled plasma atomic emission spectroscopy
FTIR	Fourier Transform Infrared Spectroscopy
XPS	X-ray photoelectron spectroscopy
TGA	Thermal gravimetric analysis or thermogravimetric analysis
SEM	Scanning electron microscope
SEM-EDX	Scanning electron microscopy-energy dispersive X-ray analysis
BET	Brunauer-Emmett-Teller
POC	porous organic cages
CN-APEI	Nitrile- Algal-polyethyleneimine
DrDTG	Derivative- Differential thermogravimetric analysis
SD	Sorbent dosage
PFORE	Pseudo-first order rate equation
PSORE	Pseudo-second order rate equation
RIDE	Resistance to intraparticle diffusion equation – Crank equation
SC	selectivity coefficient
DMF	Di methyl formamide
pH_PZC_	Point of zero charge
pH_0_	Initial pH of the solution
pH_eq_	Equivalent pH after sorption
K_d_	Distribution ratio
C_0_	Initial concentration
C_eq_	Equilibrium concentration after metal sorption
ICPS	Inductively coupled plasma atomic emission spectrometer
S	Sorption
D	Desorption
BEs	Binding energies
AF	Atomic Fractions
