# Supplementary material for: Amidoxime Functionalization of Algal/Polyethyleneimine Beads for the Sorption of Sr(II) from Aqueous Solutions
Source: Molecules. 2019 Oct 29;24(21):3893. doi: 10.3390/molecules24213893 (PMC6864727; doi:10.3390/molecules24213893)
Supplement: Supplementary file 1 [file molecules-24-03893-s001.pdf]

Supplementary Information

Amidoxime Functionalization of Algal/Polyethyleneimine Beads for the Sorption of Sr(II) from Aqueous Solutions

Yuezhou Wei [1], Khalid A.M. Salih [1], Siming Lu [1], Mohammed F. Hamza [1,2,3,*], Toyohisa Fujita [1], Thierry Vincent [3] and Eric Guibal [3,*]

[1] Guangxi Key Laboratory of Processing for Non-ferrous Metals and Featured Materials, School of Resources, Environment and Materials, Guangxi University, Nanning 530004, China; yzwei@gxu.edu.cn (Y.W.); m_fouda21@hotmail.com (M.F.H.); Immortaltiger7@gmail.com (K.A.M.S.); lusiming0302@icloud.com (S.L.); fujitatoyohisa@gxu.edu.cn (T.F.)

[2] Nuclear Materials Authority, POB 530, El-Maadi, Cairo, Egypt

[3] C2MA, IMT–Mines Ales, Univ. Montpellier, F-30319 Alès cedex, France; Thierry.Vincent@mines-ales.fr

* Correspondence: eric.guibal@mines-ales.fr (E.G.); m_fouda21@hotmail.com (M.F.H.).

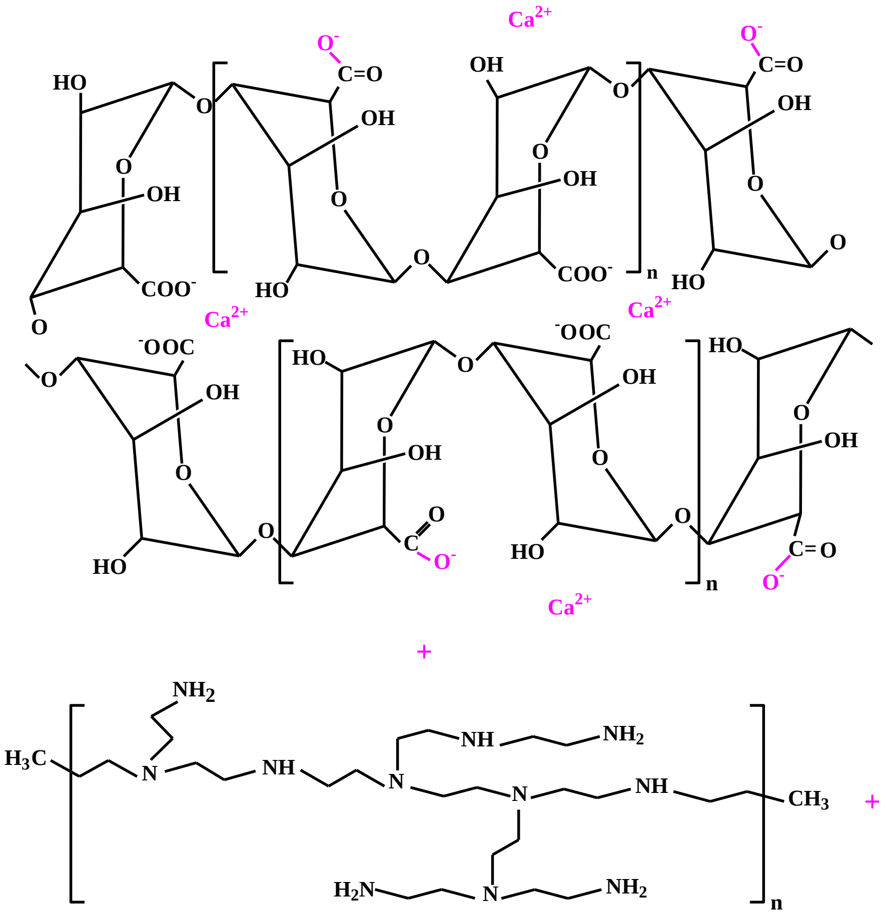

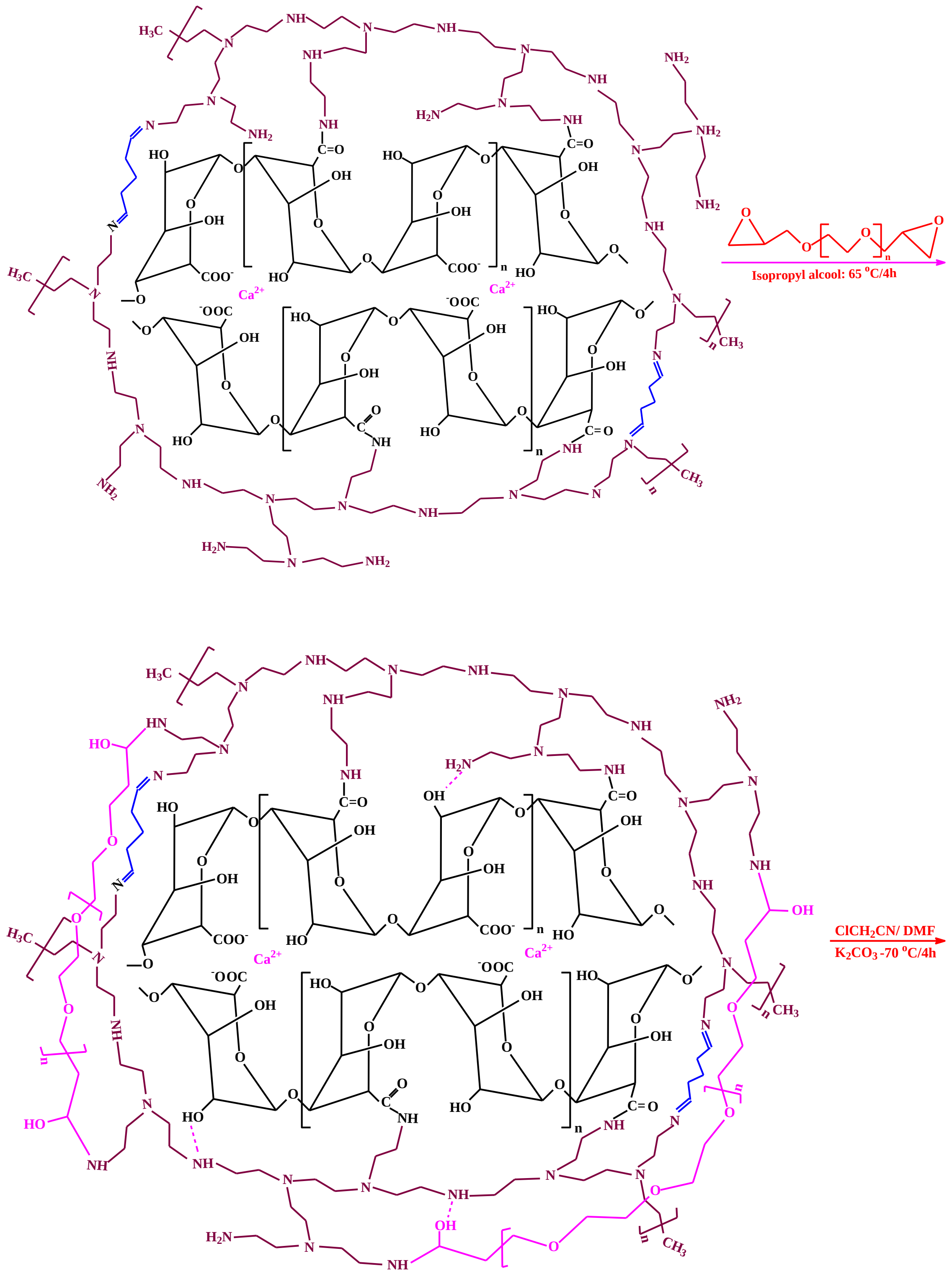
Isopropyl alcool: 65 °C/4h
ClCH2CN/ DMF
K2CO3 -70 °C/4h

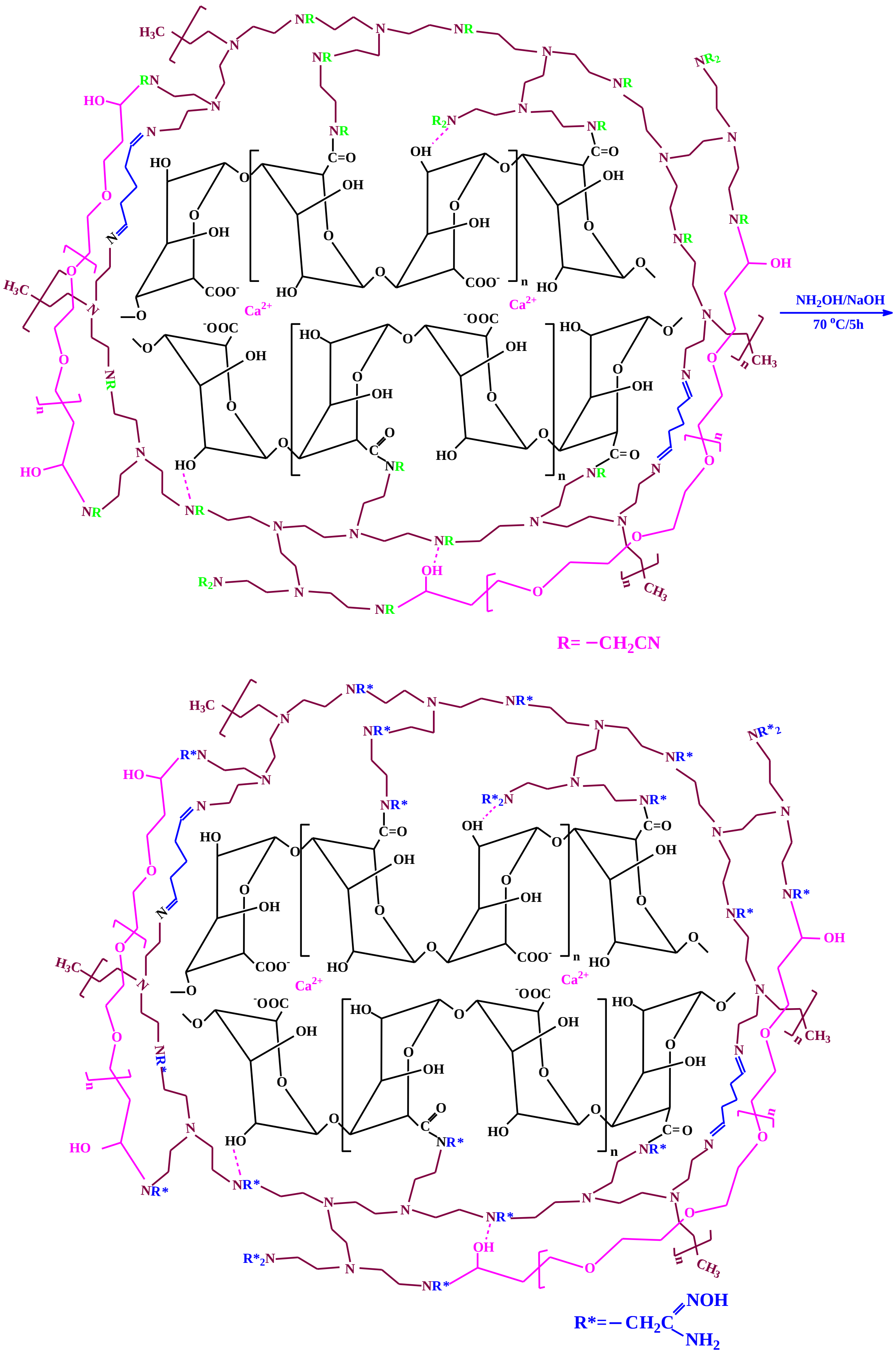

Scheme S1. Schematic presentation of the different steps involved in APEI functionalization (amidoximation).

Table S1. SEM and EDX analysis of the raw material (APEI), nitrilated material (CN-APEI), and amidoxime-functionalized sorbent (AO-APEI), before and after Sr(II) sorption, after Sr(II) desorption and after the fifth cycle of sorption and desorption, and after treatment with sodium chloride for either raw materials and AO sorbent.

Material	SEM analysis	EDX analysis
APEI	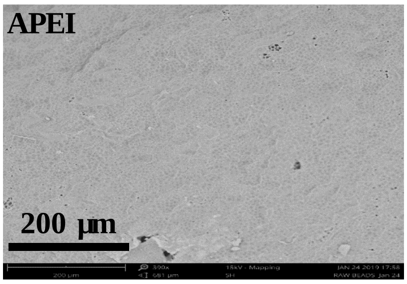	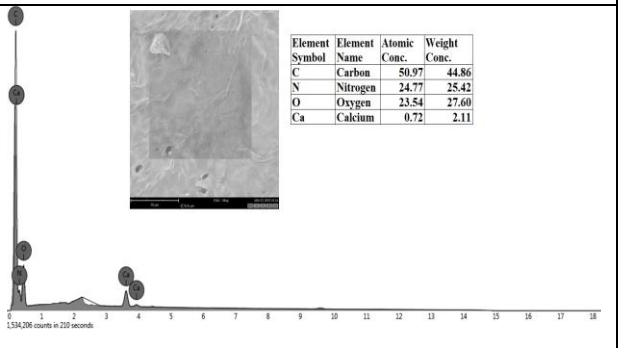
CN-APEI	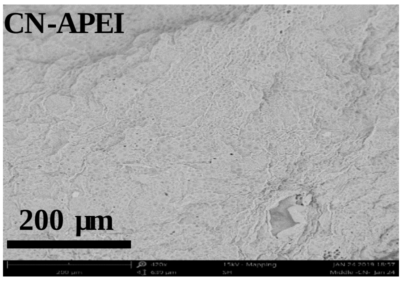	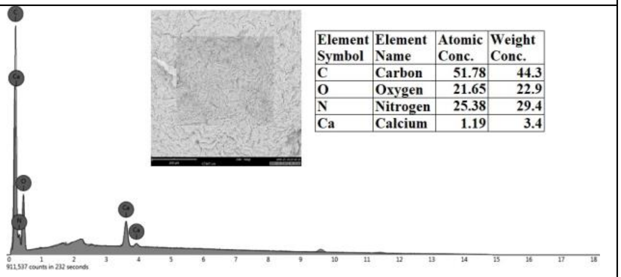
AO-APEI	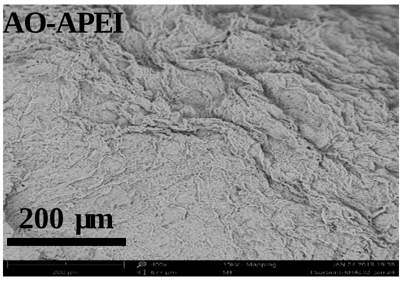	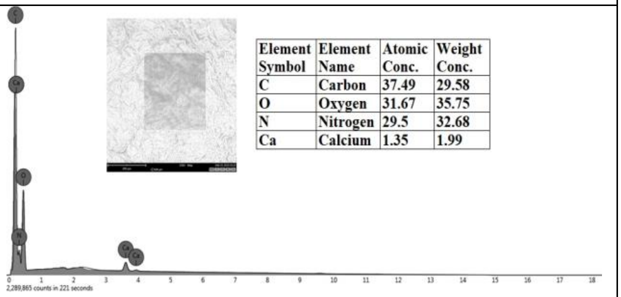
AO-APEI + Sr(II)	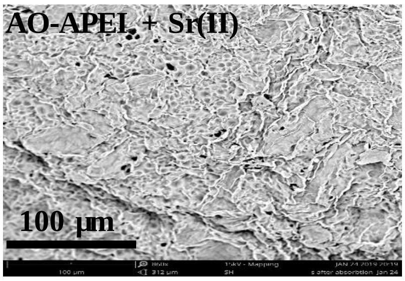	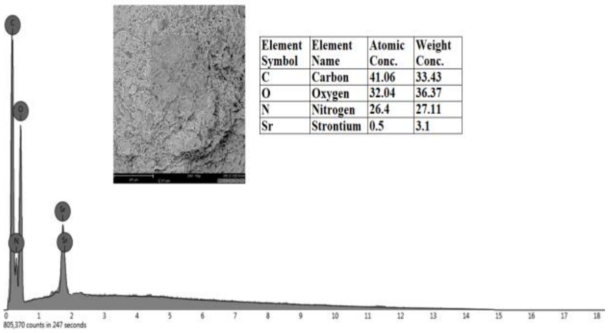

<table>
<tr>
<td>AO-APEI after desorption</td>
<td>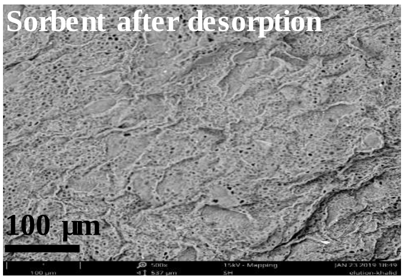
</td>
<td>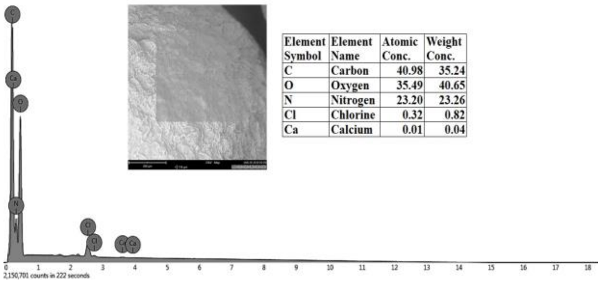
</td>
</tr>
<tr>
<td>AO-APEI after 5 cycles</td>
<td>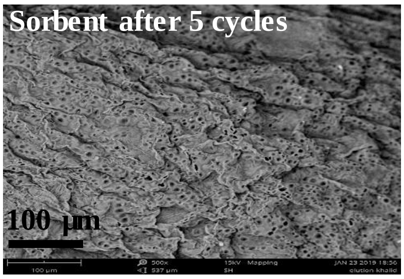
</td>
<td>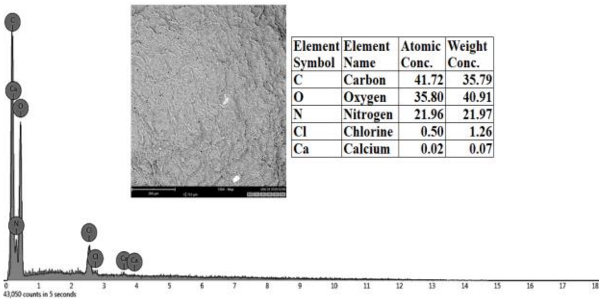
</td>
</tr>
<tr>
<td>APEI after exposure to NaCl solution</td>
<td>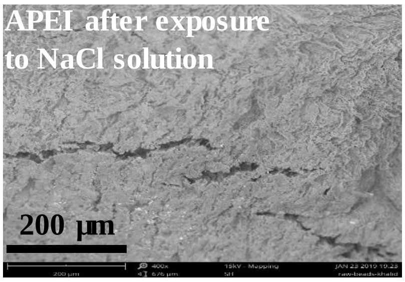
</td>
<td>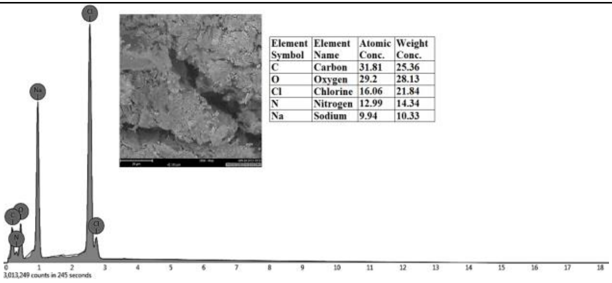
</td>
</tr>
<tr>
<td>AO-APEI after exposure to NaCl solution</td>
<td>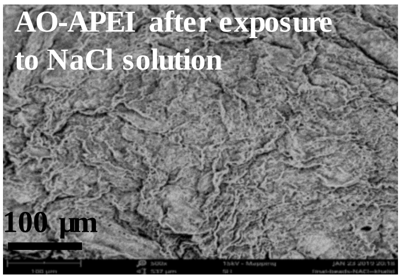
</td>
<td>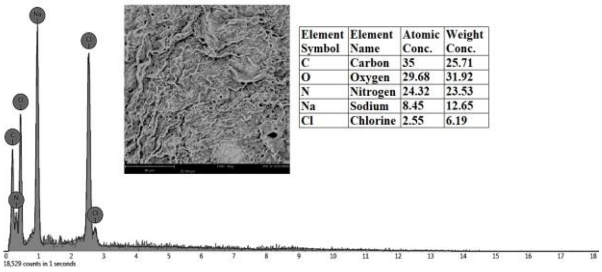
</td>
</tr>
</table>

Table S2. Assignments and characteristic wavenumbers (Wn, cm^{-1}) of the different peaks of the spectra of sorbent (at the different steps of the synthesis, after metal sorption and after 5 recycling cycles).

Vibration	Ref.	Wn. in ref.	APEI	CN-APEI	AO-APEI	AO-APEI + Sr(II)	AO-APEI after desorption	AO-APEI (5 cycles)
O-H overlapped with N-H stretching	[1, 2]	3500 -3000	3421	3419	3423	3423	3423	3419
C≡N Stretching (nitrile group)	[3]	2280-2240	==	2198	==	==	==	==
C=N and C=O Stretching (amide)	[3, 4]	1690-1630	1629	1604	1612	1619	1620	1621
(C-N) of -CO-NH_2 (primary and secondary) and-OH Bending	[5, 6]	1450-1330	1411	1411	1409	**	1408	1406
C-N Stretching + symmetric C-O Stretching	[3, 6-9]	1342-1030	1097	1093	1084	1101	1085	1089
C-N Stretching (primary amine) + C-O-C Asymmetric Stretching	[8, 10, 11]	1090-1020	1037	1032	1032	1018	1033	1024

Table S3. XPS characterization of APEI and CN-APEI materials.

<table>
<tr><td>Signal</td><td>APEI</td><td>CN-APEI</td></tr>
<tr><td>C 1S</td><td>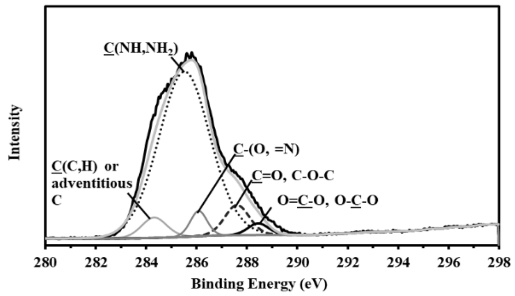
</td><td>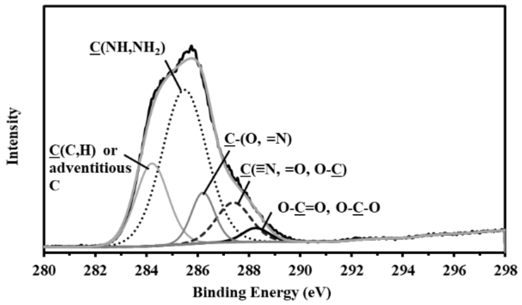
</td></tr>
<tr><td>O 1s</td><td>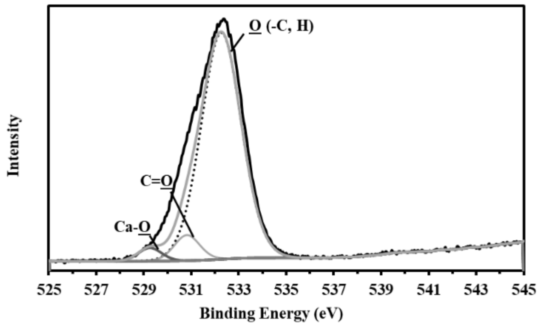
</td><td>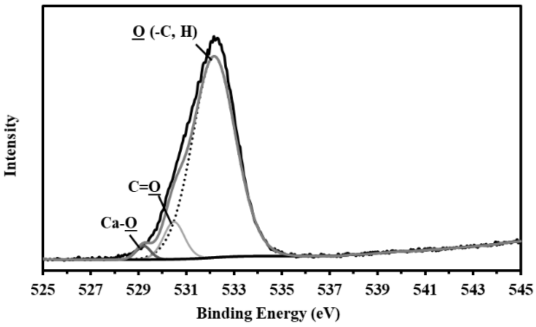
</td></tr>
<tr><td>N 1s</td><td>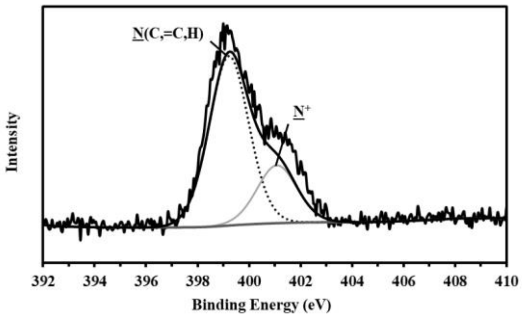
</td><td>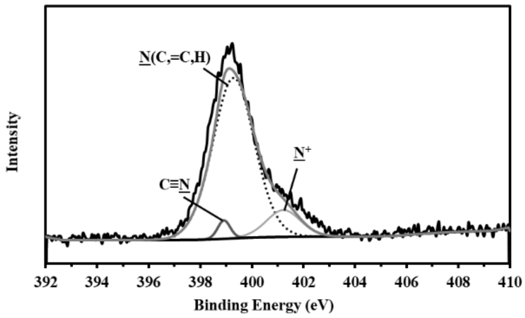
</td></tr>
<tr><td>S 2p</td><td>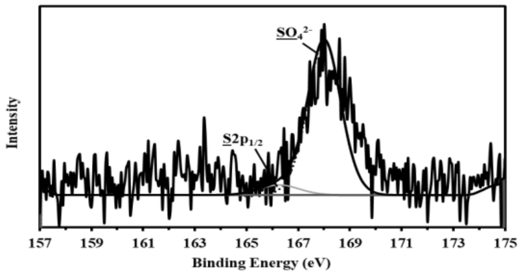
</td><td>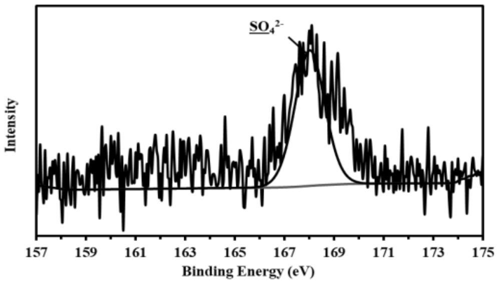
</td></tr>
<tr><td>Ca 2p</td><td>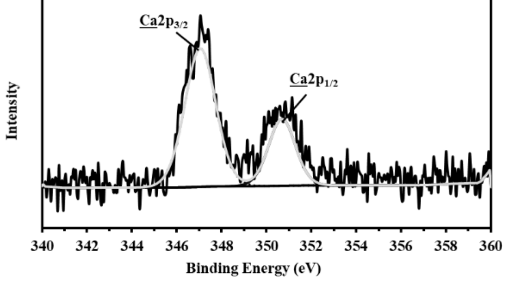
</td><td>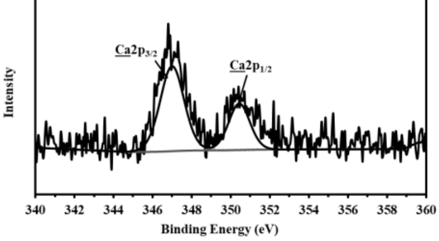
</td></tr>
</table>

Table S4. XPS characterization of AO-APEI before and after Sr(II) sorption

Signal	AO-APEI	AO-APEI + Sr(II)
C *1S*	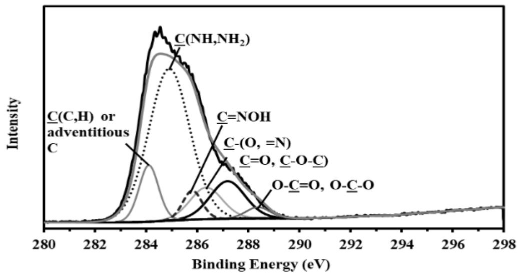	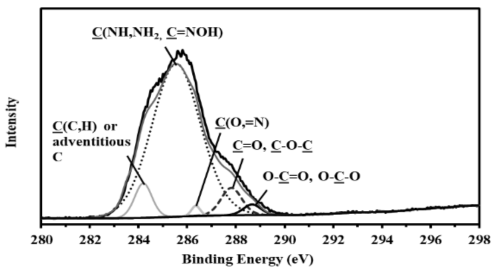
O *1s*	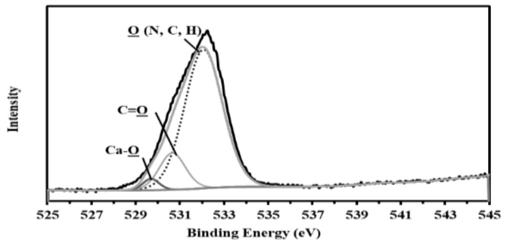	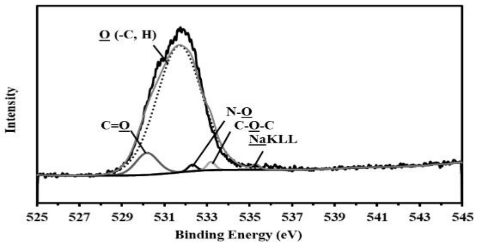
N *1s*	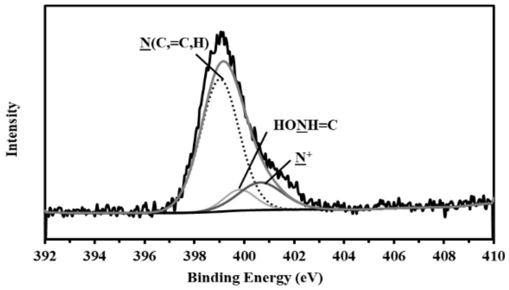	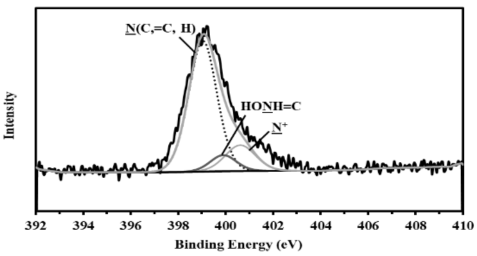
S *2p*<br>*&*<br>Na *1s*	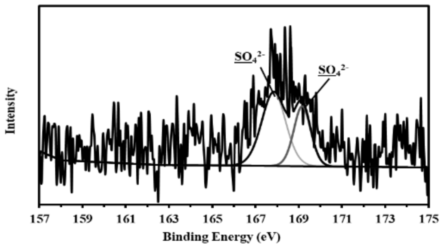	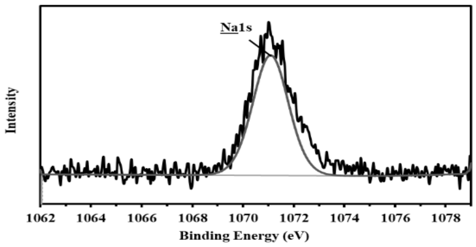
Ca *2p*	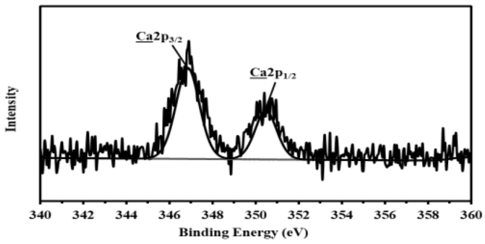	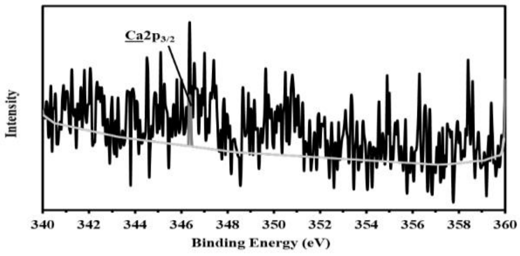
Sr *3d*		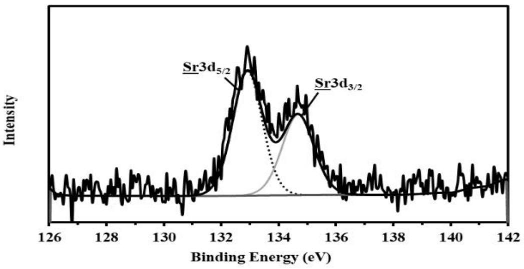

Table S5. Assignments, Binding Energies (BEs) and Atomic Fractions (AF, %) on the different stages of synthesis and after sorption.

Signal	APEI	CN-APEI	AO-APEI	AO-APEI + Sr(II)	Assignments
	BE (eV) (AF ,%)	BE (eV) (AF, %)	BE (eV) (AF, %)	BE (eV) (AF, %)	
C 1*s*	284.27 (4.94)	284.27 (20.31)	284.1 (10.94)	284.25 (10.55)	C-C , C-H or adventitious C
	285.41 (84.6)	285.43 (50.28)	284.9 (61.59)	285.69 (75.09)	C-NH or C-NH_2
			285.6 (5.5)		-C=NOH
	286.08 (2.71)	286.1 (12.48)	286.35 (10.19)	286.18 (0.85)	C-O, C=N
	287.6 (5.7)	287.32 (14.06)	287.62 (8.58)	287.85 (9.42)	C=O, C-O-C + *(C≡N for nitrile)*
	288.44 (2.05)	288.25 (2.87)	288.8 (3.2)	288.95 (2.15)	O-C=O, O-C-O
O 1*s*	529.29 (4.43)	529.09 (1.87)	529.24 (1.57)	==	O-Ca
	530.7 (12.82)	530.31 (10.36)	530.65 (14.2)	530.18 (8.78)	C=O
	532.27 (82.75)	532.16 (87.77)	532.07 (84.23)	531.91 (87.22)	C-O, OH
				532.31 (1.59)	O-N
				533.19 (1.9	C-O-C
				535.4 (0.51)	Na KLL
N 1*s*	399.2 (71.76)	399.12 (62.89)	399.02 (65.43)	398.88 (93.04)	C-N, C=NH. -NH
			399.91 (19.07)		-C=NOH
	401.34 (28.24)	400.51 (33.21)	400.64 (15.5)		N^+
		398.3 (3.9)			C≡N
				405.95 (6.96)	NO_3
S 2*p*	165.98 (19.03)				S 2$p_{1/2}$
	168.01 (80.97)	168.02 (100)	167.9 (60.92), 169 (39.08)		SO_4^{2-}
Ca 2*p*	347.12 (64.41)	346.97 (63.18)	346.89 (61.99)	346.37 (100) very weak	Ca 2$p_{3/2}$
	350.69 (35.59)	350.34 (36.82)	350.4 (38.01)	==	Ca 2$p_{1/2}$
Sr 3*d*				132.91 (54.63)	Sr 3$d_{5/2}$
				134.59 (45.37)	Sr 3$d_{3/2}$

Table S6. Sorption capacities (q_{eq}, µmol g^{-1}) and distribution ratios (K_d, L kg^{-1}) for metal sorption from multi-metal equimolar concentrations at different equilibrium pH values.

pH_{eq}	Mg(II)		Al(III)		Ca(II)		Na(I)		Sr(II)		Cumul.
	q_{eq}	K_d	q_{eq}	K_d	q_{eq}	K_d	q_{eq}	K_d	q_{eq}	K_d	$q_{m,cumul}$
2.30	105.6	60.6	113.2	66.9	26.3	15.3	50.6	32.3	898.7	1407	1194
3.19	385.3	280.9	246.4	162.9	57.0	34.0	108.0	72.5	933.1	1646	1730
4.82	459.0	353.0	310.6	214.8	46.9	27.7	225.8	167.5	1134.0	3102	2176
5.39	507.9	411.2	425.6	327.9	395.8	317.4	374.2	322.7	1294.3	8387	2998
7.02	622.8	574.0	423.8	326.8	565.4	549.8	531.1	554.4	1382.2	42287	3525
7.62	629.6	586.7	476.5	388.1	645.3	698.7	555.9	601.9	1367.6	30740	3675

Table S7. SEM and EDX analysis of AO-APEI sorbent after loading with multi-metal solutions (Ca(II), Na(I), Mg(II), Al(III) and Sr(II)) at different pH values (i.e., 2-7)

SEM analysis	EDX analysis

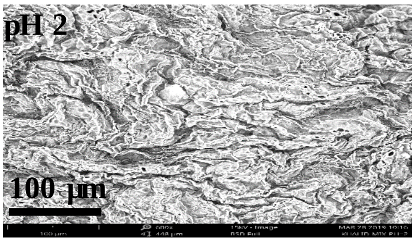

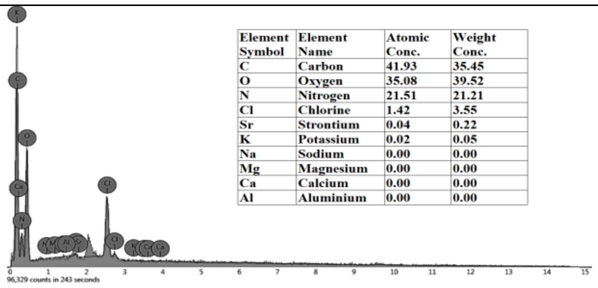

Element Symbol	Element Name	Atomic Conc.	Weight Conc.
C	Carbon	41.93	35.45
O	Oxygen	35.08	39.52
N	Nitrogen	21.51	21.21
Cl	Chlorine	1.42	3.55
Sr	Strontium	0.04	0.22
K	Potassium	0.02	0.05
Na	Sodium	0.00	0.00
Mg	Magnesium	0.00	0.00
Ca	Calcium	0.00	0.00
Al	Aluminium	0.00	0.00

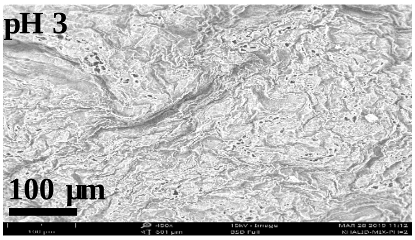

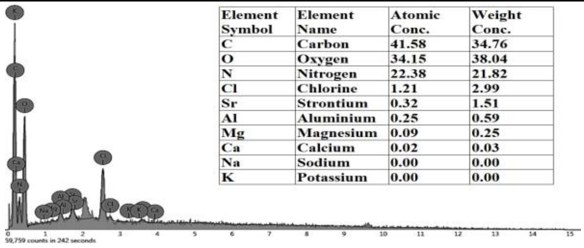

Element Symbol	Element Name	Atomic Conc.	Weight Conc.
C	Carbon	41.58	34.76
O	Oxygen	34.15	38.04
N	Nitrogen	22.38	21.82
Cl	Chlorine	1.21	2.99
Sr	Strontium	0.32	1.51
Al	Aluminium	0.25	0.59
Mg	Magnesium	0.09	0.25
Ca	Calcium	0.02	0.03
Na	Sodium	0.00	0.00
K	Potassium	0.00	0.00

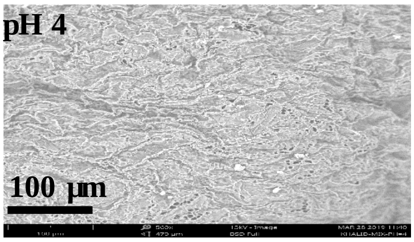

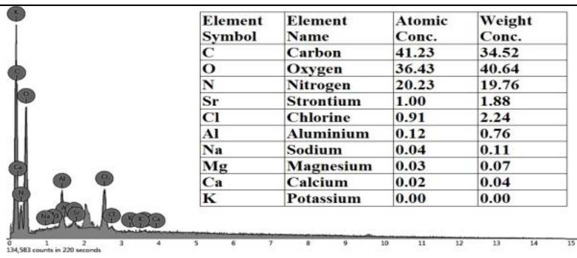

Element Symbol	Element Name	Atomic Conc.	Weight Conc.
C	Carbon	41.23	34.52
O	Oxygen	36.43	40.64
N	Nitrogen	20.23	19.76
Sr	Strontium	1.00	1.88
Cl	Chlorine	0.91	2.24
Al	Aluminium	0.12	0.76
Na	Sodium	0.04	0.11
Mg	Magnesium	0.03	0.07
Ca	Calcium	0.02	0.04
K	Potassium	0.00	0.00

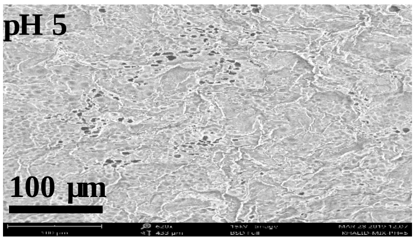

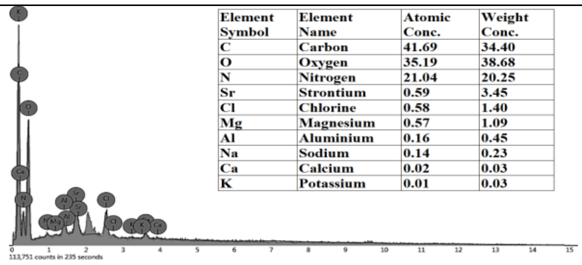

Element Symbol	Element Name	Atomic Conc.	Weight Conc.
C	Carbon	41.69	34.40
O	Oxygen	35.19	38.68
N	Nitrogen	21.04	20.25
Sr	Strontium	0.59	3.45
Cl	Chlorine	0.58	1.40
Mg	Magnesium	0.57	1.09
Al	Aluminium	0.16	0.45
Na	Sodium	0.14	0.23
Ca	Calcium	0.02	0.03
K	Potassium	0.01	0.03

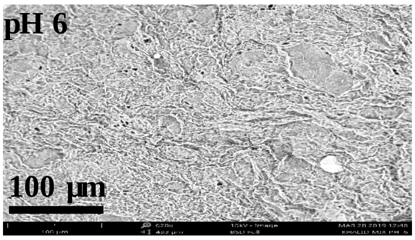

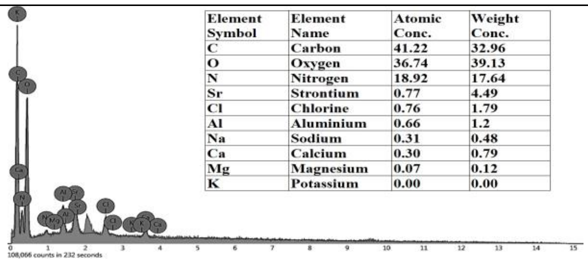

Element Symbol	Element Name	Atomic Conc.	Weight Conc.
C	Carbon	41.22	32.96
O	Oxygen	36.74	39.13
N	Nitrogen	18.92	17.64
Sr	Strontium	0.77	4.49
Cl	Chlorine	0.76	1.79
Al	Aluminium	0.66	1.2
Na	Sodium	0.31	0.48
Ca	Calcium	0.30	0.79
Mg	Magnesium	0.07	0.12
K	Potassium	0.00	0.00

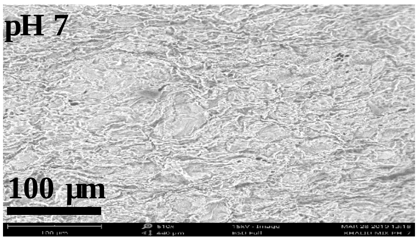

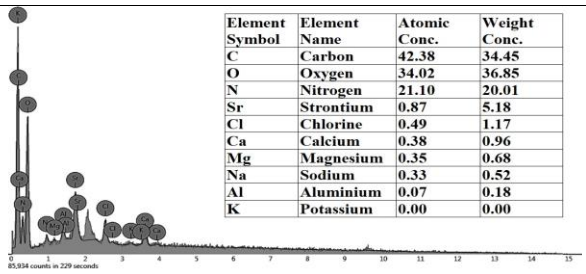

Element Symbol	Element Name	Atomic Conc.	Weight Conc.
C	Carbon	42.38	34.45
O	Oxygen	34.02	36.85
N	Nitrogen	21.10	20.01
Sr	Strontium	0.87	5.18
Cl	Chlorine	0.49	1.17
Ca	Calcium	0.38	0.96
Mg	Magnesium	0.35	0.68
Na	Sodium	0.33	0.52
Al	Aluminium	0.07	0.18
K	Potassium	0.00	0.00

Table S8. Modeling of breakthrough curves for the sorption of Sr(II) using AO-PEI – Parameters of the Thomas model [12].

Flow rate (mL min^{-1})	$q_{th.}$ (mg Sr g^{-1})	$q_{exp.}$ (mg Sr g^{-1})	q_{model} (mg Sr g^{-1})	$k_T \times 10^3$ (L mg^{-1} h^{-1})	R_2
0.2 (0.012)[a]		79.3	81.4	5.52	0.975
0.4 (0.024)[a]	114.5	66.6	67.8	21.0	0.986
1.0 (0.060)[a]		50.6	52.2	74.8	0.988

(a) Flow rate (L h^{-1})

Thomas equation:

$$\ln\left(\frac{C_0}{C(t)} - 1\right) = \frac{k_T\, q_{eq} m}{Q} - k_T C_0\, t$$

Where k_T is the Thomas rate constant (L mg^{-1} h^{-1}), q_{eq} the sorption capacity at equilibrium (mg Sr g^{-1}), m the amount of sorbent (g), Q the flow rate (L h^{-1}); t the time (h), C_0 and C(t): the inlet and outlet concentrations, respectively.

Table S9. Modeling of kinetic profiles for Sr(II) desorption from metal-loaded AO-PEI – PFORE and PSORE equations [13].

Model	PFORE		PSORE		
Parameter	k_{D1} (min^{-1})	R^2	β_2	k_{D2} (min^{-1})	R^2
Value	0.098	0.986	0.989	0.198	0.983

PFORE: $\frac{q(t)}{q_0} = e^{-k_{D1}t}$ with: k_{D1} the apparent rate coefficient for desorption (min^{-1}) PSORE: $\frac{q(t)}{q_0} = \frac{1}{\beta_2 + k_{D2}t}$ with: k_{D2} the apparent rate coefficient for desorption (min^{-1}) and β_2 (dimension less) the constant for PSORE (in desorption).

Table S10. Summary of the results obtained in the treatment of two seawater samples (after 18 h of contact).

	Beihai seawater sample				Da Nan seawater sample			
Element	C_0 (mg L^{-1})	C_{18h} (mg L^{-1})	q_{18h} (mmol g^{-1})	K_d (L g^{-1})	C_0 (mg L^{-1})	C_{18h} (mg L^{-1})	q_{18h} (mmol g^{-1})	K_d (L g^{-1})
Na	11506	11472	16.7	0.034	12612	12511	22.4	0.042
K	488	483	0.742	0.061	513	512	0.767	0.059
Mg	1299	1293	1.364	0.026	1224	1220	1.035	0.021
Ca	455	452	0.339	0.031	442	439	0.431	0.040
Sr	4.36	1.01	0.191	16.55	4.69	1.06	0.207	17.12
B	2.85	1.45	0.647	4.80	3.99	2.09	0.868	4.52
As	0.09	0.029	0.0040	10.3	0.04	0.018	0.0015	6.22

(Experimental conditions: SD = 0.2 g L^{-1}; Time: 18 h; Temperature: 25 °C; agitation speed: 140 rpm).

Table S11. Semi-quantitative EDX analysis of sorbent (surface and interior; average of 4 or 5 analyses (weight percentage) with standard deviation into parentheses) after treatment of two seawater samples (Beihai, China and Da Nan, Vietnam).

Element	Beihai seawater sample		Da Nan seawater sample	
	Surface	Interior	Surface	Interior
Carbon	33.80 (± 1.76)	35.74 (± 0.54)	34.87 (± 1.79)	36.32 (± 1.14)
Boron	20.22 (± 0.69)	20.28 (± 0.28)	19.56 (± 0.50)	19.32 (± 0.56)
Oxygen	17.85 (± 1.30)	20.68 (± 0.44)	19.19 (± 1.07)	20.53 (± 1.27)
Nitrogen	11.29 (± 0.90)	13.05 (± 0.46)	11.43 (± 0.90)	13.31 (± 0.34)
Sodium	2.95 (± 0.53)	2.11 (± 0.62)	2.63 (± 0.25)	1.79 (± 0.25)
Chlorine	6.53 (± 1.17)	2.90 (± 0.41)	5.34 (± 0.88)	3.01 (± 0.18)
Calcium	1.75 (± 0.60)	0.57 (± 0.04)	1.46 (± 0.38)	0.73 (± 0.05)
Sulfur	0.93 (± 0.17)	0.40 (± 0.06)	0.93 (± 0.06)	0.44 (± 0.06)
Magnesium	0.47 (± 0.08)	0.46 (± 0.04)	0.42 (± 0.07)	0.49 (± 0.07)
Strontium	0.91 (± 0.33)	1.09 (± 0.18)	1.20 (± 0.40)	1.02 (± 0.20)
Potassium	0.30 (± 0.12)	0.10 (± 0.01)	0.27 (± 0.12)	0.18 (± 0.05)
Phosphorus	0.15 (± 0.04)	0.13 (± 0.01)	0.13 (± 0.03)	0.13 (± 0.01)
Uranium	1.23 (± 0.98)	1.27 (± 0.12)	0.84 (± 0.52)	1.43 (± 0.06)
Silicon	0.16 (± 0.03)	0.12 (± 0.05)	-	0.11 (± 0.06)
Cesium	1.34 (± 0.69)	0.54 (± 0.03)	1.32 (± 0.04)	0.17 (± 0.02)
Aluminium	-	0.17 (± 0.02)	-	0.17 (± 0.02)
Rubidium	-	0.37	-	0.42
Arsenic	-	0.31	-	0.19
Selenium	-	0.31	-	0.29
Scandium	-	0.14	-	0.18

- : not detected/analyzed; data without standard deviation correspond to single measurements.

Table S12. XPS characterization of APEI before and after exposure to 2 M NaCl solution for 48 h.

Signal	APEI	APEI (NaCl)
C *1S*	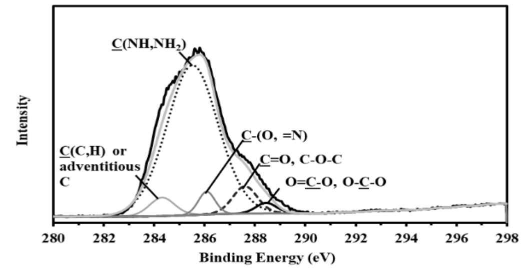	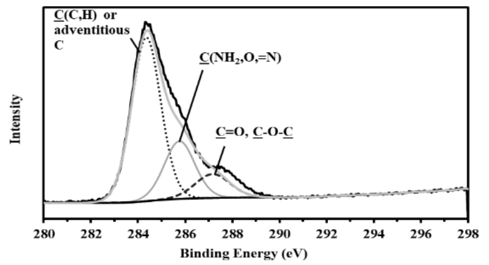
O *1s*	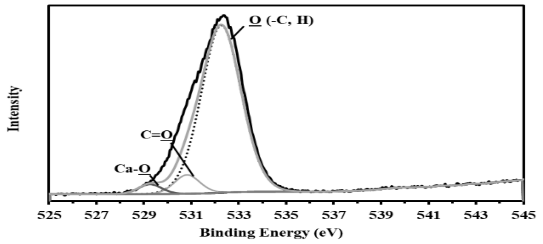	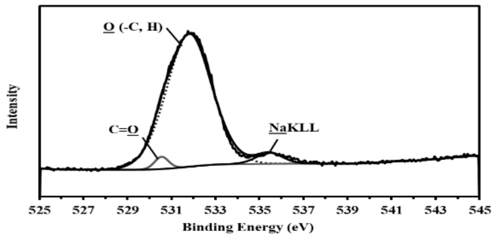
N *1s*	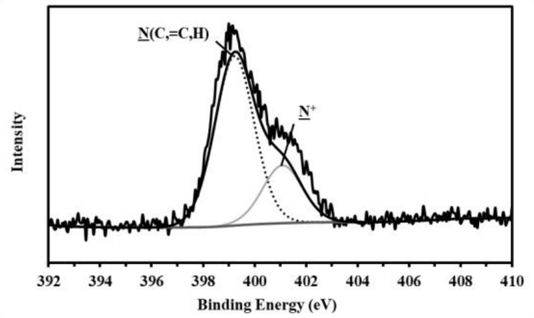	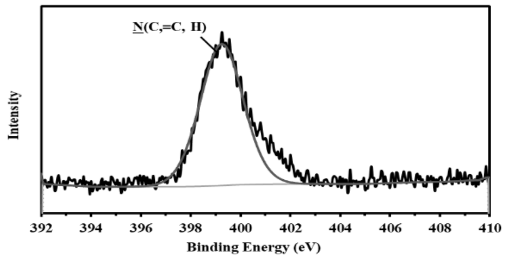
S *2p*<br>Na *1s*		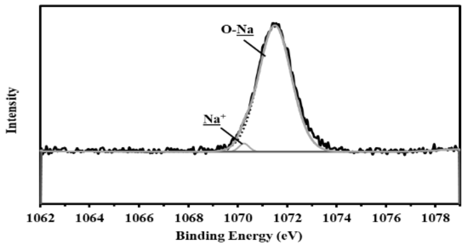
Cl *2p*		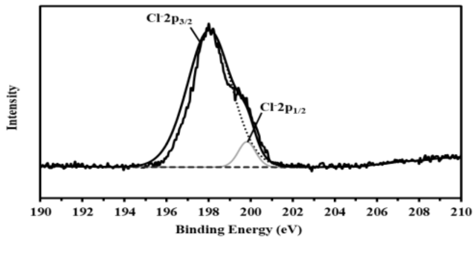
Ca *2p*	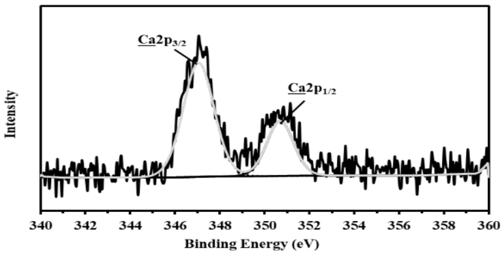	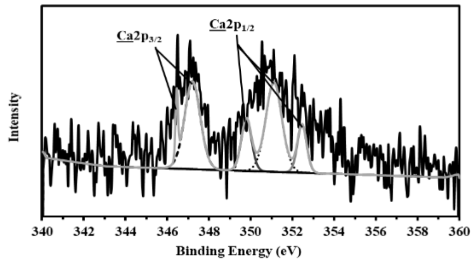

Table S13. XPS characterization of AO-APEI before and after exposure to 2 M NaCl solution for 48 h.

Signal	AO-APEI	AO-APEI (NaCl)
C *1s*	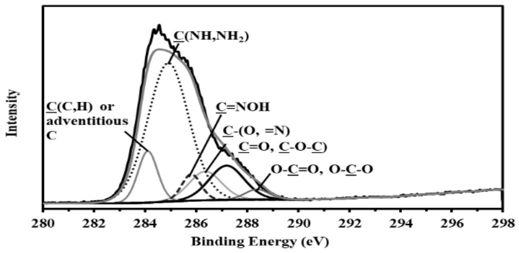	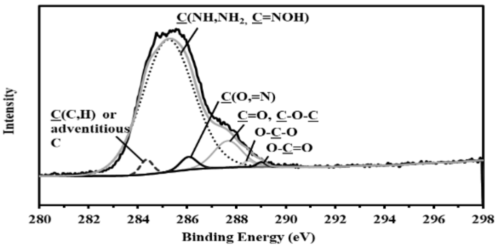
O *1s*	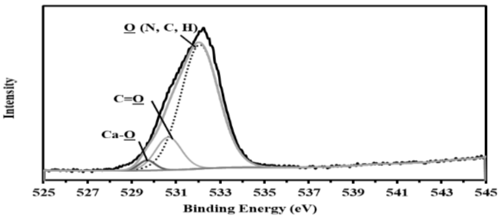	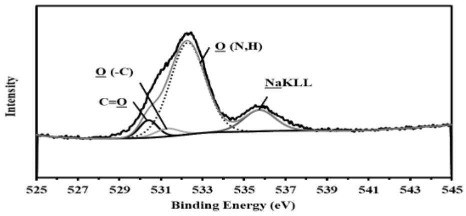
N *1s*	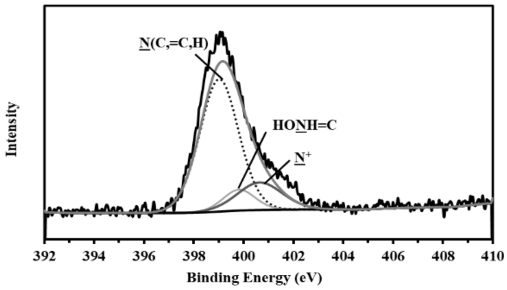	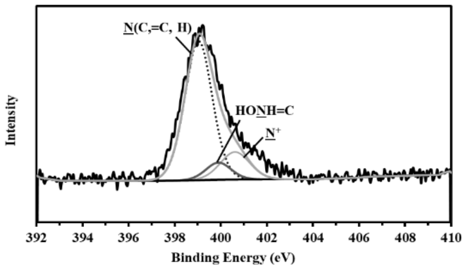
Na *1s*		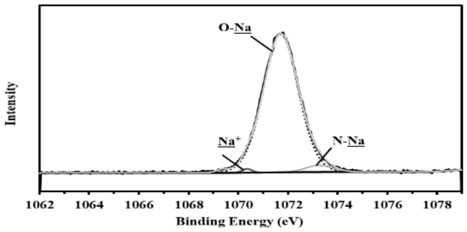
Ca *2p*	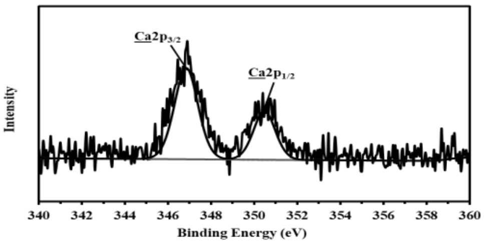	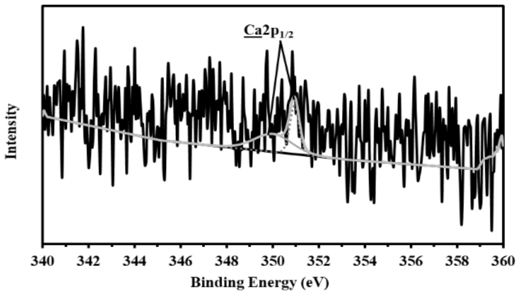
Cl *2p*		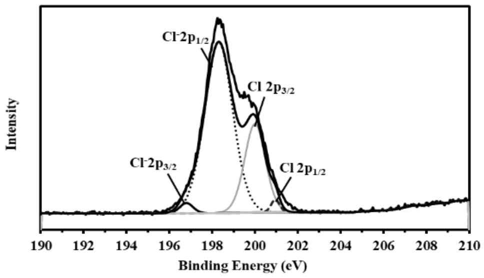

Table S14. Assignments, Binding energies (BEs) and Atomic Fractions (AF, %) of APEI and AO-APEI before and after exposure to 2 M NaCl solution for 48 h.

Signal	APEI	APEI (NaCl)	AO-APEI	AO-APEI (NaCl)	Assignments
	BE (eV) (AF, %)	BE (eV) (AF, %)	BE (eV) (AF, %)	BE (eV) (AF, %)	
C *1s*	284.27 (4.94)	284.45 (80.26)	284.1 (10.94)	284.35 (2.08)	C-C , C-H or adventitious C
	285.41 (84.6)	286.0 (7.8)	284.9 (61.59)	285.58 (85.75)*	C-NH or C-NH_2
	286.08 (2.71)	286.0 (7.8)*	286.35 (10.19)	286.15 (2.39)	C-O, C=N
	287.6 (5.7)	287.4 (11.94)	287.62 (8.58)	287.65 (8.55)	C=O, C-O-C
	288.44 (2.05)	==	288.8 (3.2)*	288.7 (0.77)	O-C-O
			285.6 (5.5)	285.58 (85.75)*	-C=NOH
			288.8 (3.2)*	289 (0.47)	O-C=O
O *1s*	529.29 (4.43	==	529.24 (1.57)	==	O-Ca
	530.7 (12.82)	530.55 (2.67)	530.65 (14.2)	530.4 (5.56)	C=O
	532.27 (82.75)	531.8 (92.76)	532.07 (84.23)*	531.19 (1.38)	C-O
		==	532.07 (84.23)*	532.22 (79.59)	O-N, OH
		==		==	C-O-C
		535.45 (4.57)		535.95 (13.47)	Na KLL
N *1s*	399.2 (71.76)	399.24 (100)	399.02 (65.43)	399.07 (69.12)	C=NH. -NH
	401.34 (28.24)	==	400.64 (15.5)	400.65 (27.03)	O=C-NH, N^+
		==	399.91 (19.07)	399.81 (3.85)	C=NOH
Na *2s*		1070.25 (1.96)		1070.35 (1.44)	Na^+
		1071.5 (98.04)		1071.71 (95.09)	O-Na
		==		1073.25 (3.47)	N-Na
Ca 2p	347.12 (64.41)	346.52(4.7), 347.1(34.33)	346.89 (61.99)		Ca $2p_{3/2}$
	350.69 (35.59)	349.95 (13.7) 351.1 (33.51) 352.45 (13.77)	350.4 (38.01)	350 (87.33), 350.87(12.67)	Ca $2p_{1/2}$
Cl *2p*		198.03 (90.97)		198.41 (71.57)	Cl $2p_{3/2}$
		199.8 (9.03)		200 (25.3)	Cl $2p_{1/2}$
		==		200.95 (1.37)	Cl $2p_{3/2}$
		==		196.8 (1.76)	Cl $2p_{1/2}$

*= overlapping binding energies

Table S15. Macroscopic morphology of APEI and AO-APEI beads and size measurements.

APEI		AO-APEI	
SEM measurements	Vernier caliper measurement	SEM measurements	Vernier caliper measurement
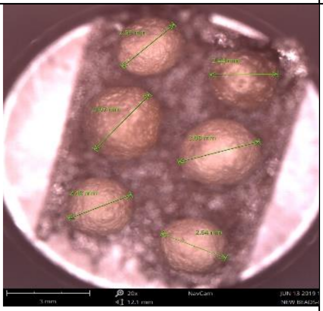	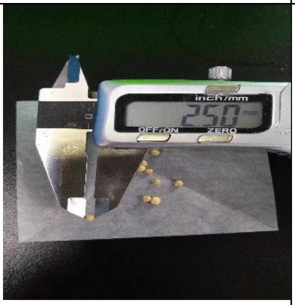	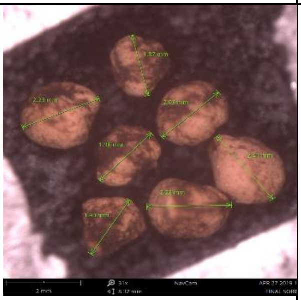	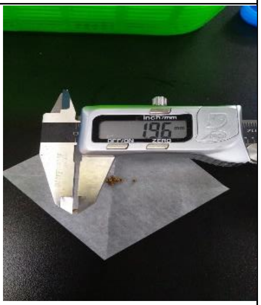
*Average of SEM measurements<br>=2.7 mm<br>*Average of Vernier caliper measurements<br>=2.76 mm	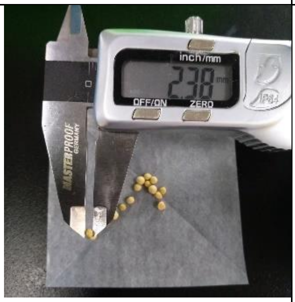	*Average of SEM measurements =2.104 mm<br>*Average of Vernier caliper measurements<br>=2.18 mm	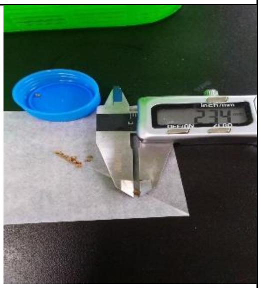
	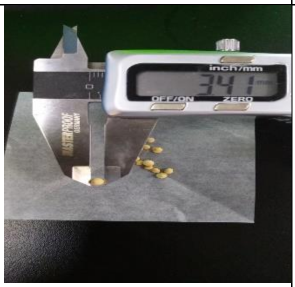		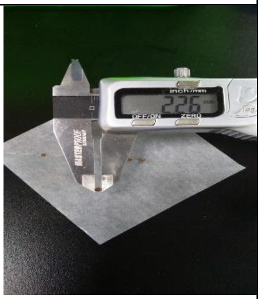

Note: Decreasing of the size is related to the type of drying; i.e., Raw beads (APEI) were freeze-dried while Q-APEI beads were air-dried.

Modeling of sorption processes

Table S16. Uptake kinetics models – PFORE (pseudo-first order rate equation), PSORE (pseudo-second order rate equation) and RIDE (resistance to intraparticle diffusion equation – Crank equation).

Model	Equation	Parameters	
PFORE [14	$q(t) = q_{eq,1}(1 - e^{k_1 t})$	$q_{eq,1}$ (mg g^{-1})	k_1 (min^{-1})
PSORE [14]	$q(t) = \dfrac{q_{eq,2}^2 \times k_2 \times t}{1 + q_{eq,2} \times k_2 \times t}$	$q_{eq,2}$ (mg g^{-1})	k_2 (L mg^{-1} min^{-1})
RIDE [15]	$\dfrac{q(t)}{q_{eq}} = 1 - \sum_{n=1}^{\infty} \dfrac{6\alpha(\alpha+1)\exp\left(\dfrac{-D_e q_n^2 t}{r^2}\right)}{9 + 9\alpha + q_n^2\alpha^2}$ With q_n being the non-zero roots of $\tan q_n = \dfrac{3 q_n}{3 + \alpha q_n^2}$ and $\dfrac{m q}{V C_o} = \dfrac{1}{1+\alpha}$	D_e (m^2 min^{-1})	

Table S17. Sorption isotherm models [16, 17]

Model	Langmuir	Freundlich	Sips
Equation	$q = \dfrac{q_{m,L} \times b_L \times C_{eq}}{1 + b_L \times C_{eq}}$	$q = k_F\, C_{eq}^{1/n}$	$q = \dfrac{q_{m,S} \times b_S \times C_{eq\,eq}^{\;1/n_S}}{1 + b_S \times C_{eq\,eq}^{\;1/n_S}}$
Parameters	$q_{m,L}$ (mg g^{-1})*	k_F (mg$^{1-1/n}$ g^{-1} L$^{-1/n}$)	$q_{m,S}$ (mmol g^{-1})*
	b_L (L mg^{-1})**	n (dimensionless)	b_S (L mg^{-1})**
	-	-	n_S (dimensionless)

*: Sorption capacity at saturation of the monolayer; **: Affinity coefficient

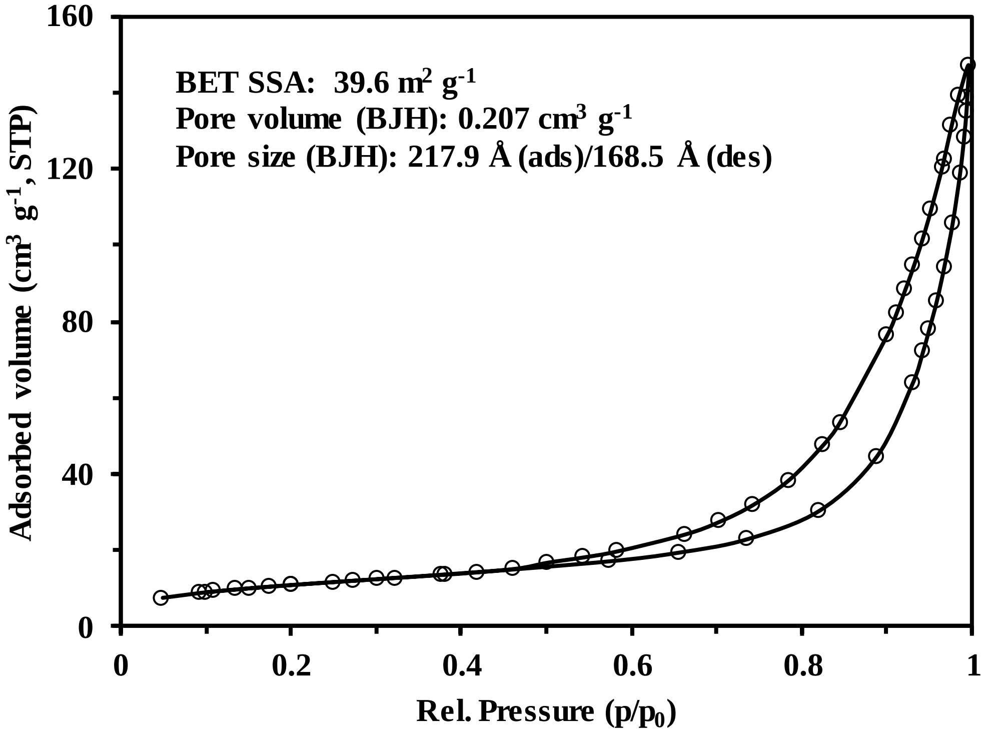

Figure 1. Textural analysis of AO-APEI sorbent.

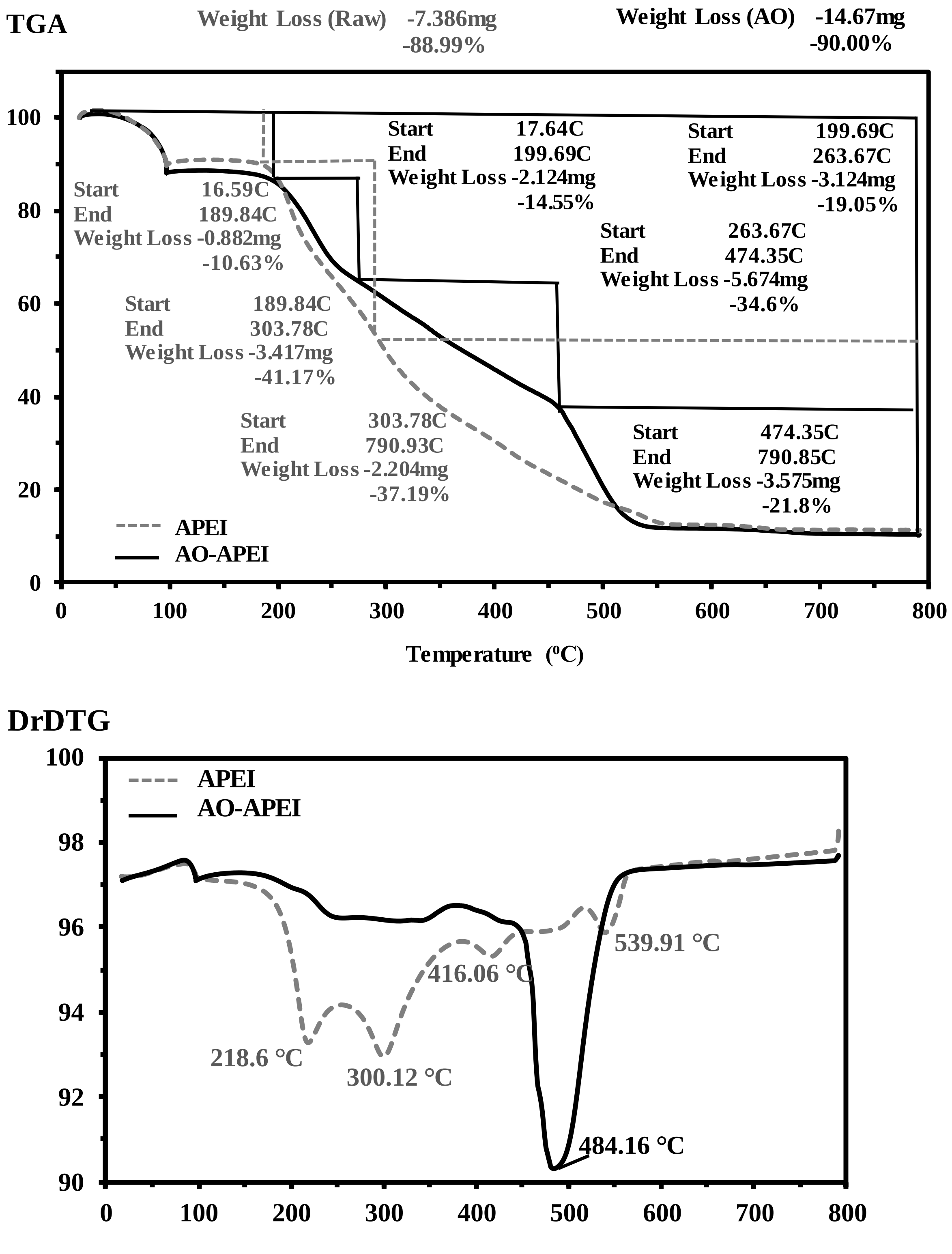

Figure S2. Thermal analysis (TGA and DrDTG) of APEI and AO-APEI sorbents (Temperature ramp: 10 °C/min; N_2 atmosphere).

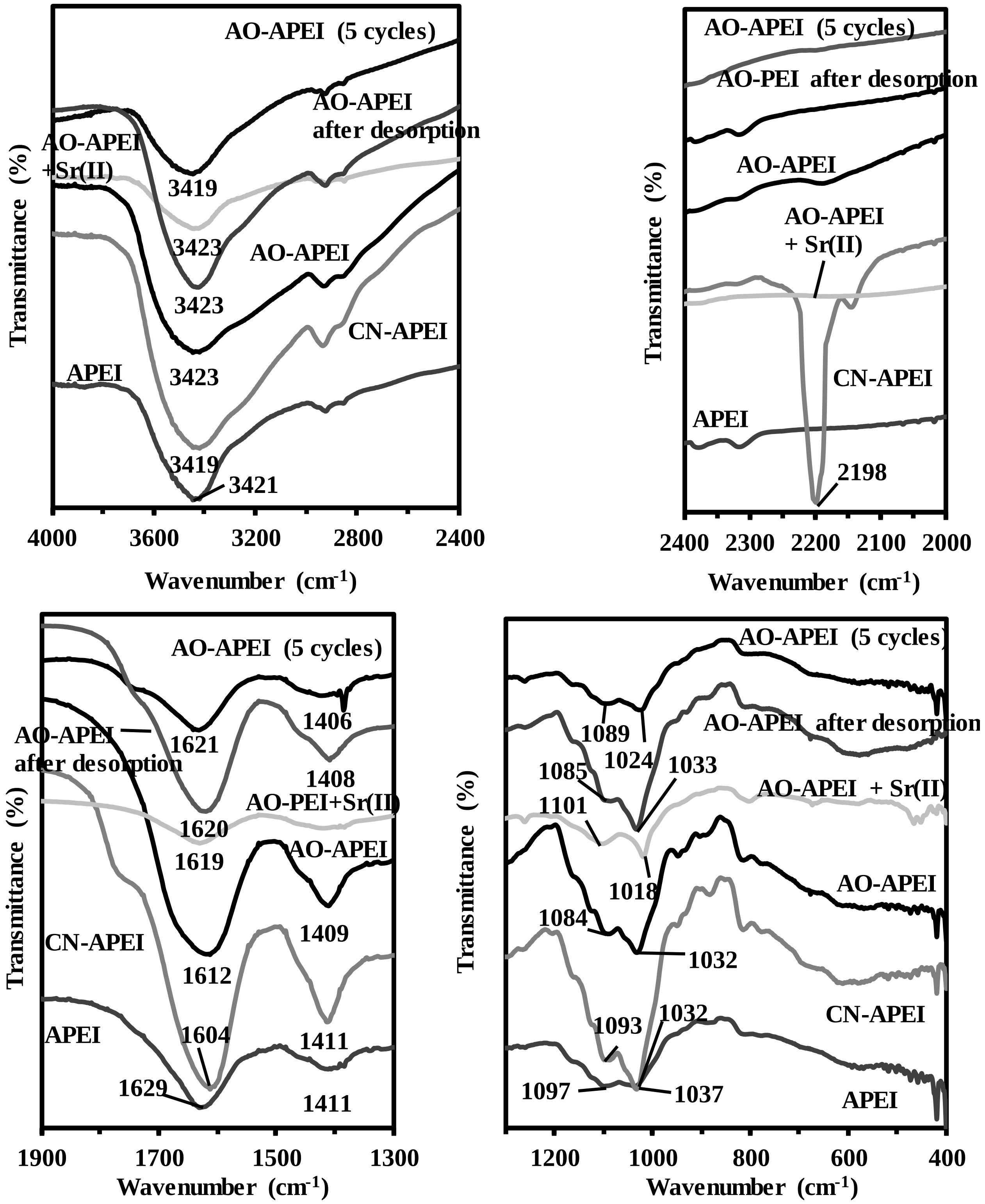

AO-APEI (5 cycles)
AO-APEI
after desorption
AO-APEI
+Sr(II)
3419
3423
3423
AO-APEI
CN-APEI
3423
APEI
3419
3421
Transmittance (%)
4000
3600
3200
2800
2400
Wavenumber (cm-1)
AO-APEI (5 cycles)
AO-PEI after desorption
AO-APEI
AO-APEI
+ Sr(II)
CN-APEI
APEI
2198
Transmittance (%)
2400
2300
2200
2100
2000
Wavenumber (cm-1)
AO-APEI (5 cycles)
AO-APEI
after desorption
1621
1406
1408
1620
AO-PEI+Sr(II)
1619
AO-APEI
CN-APEI
1409
1612
APEI
1604
1411
1629
1411
Transmittance (%)
1900
1700
1500
1300
Wavenumber (cm-1)
AO-APEI (5 cycles)
1089
AO-APEI after desorption
1024
1085
1033
AO-APEI + Sr(II)
1101
1018
AO-APEI
1084
1032
1032
CN-APEI
1093
1097
1037
APEI
Transmittance (%)
1200
1000
800
600
400
Wavenumber (cm-1)

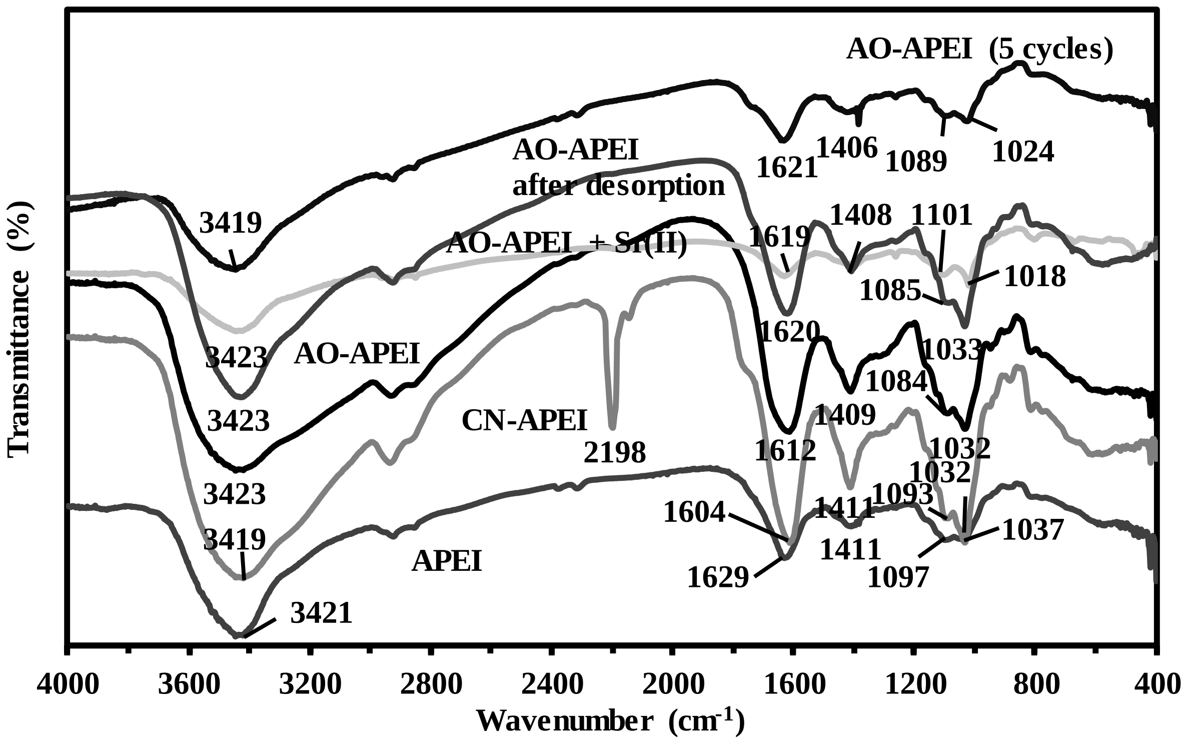

Figure S3. FTIR spectra of APEI, CN-APEI, AO-APEI materials, sorbent after Sr(II) sorption, after metal desorption and after 5 cycles of sorption and desorption (test of sorbent stability at recycling).

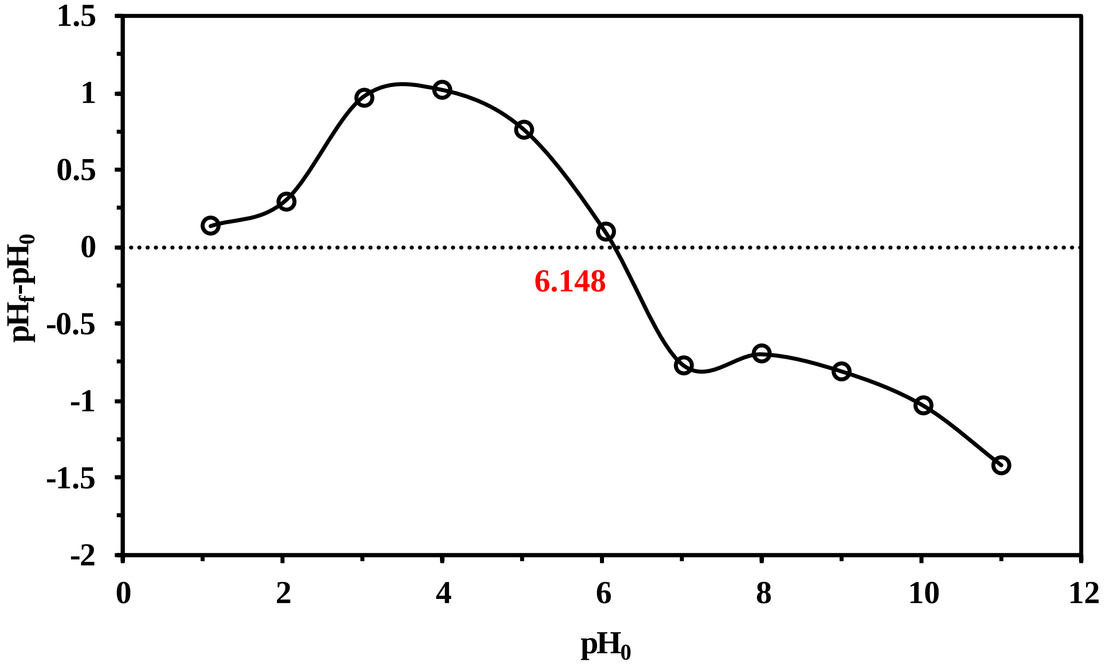

Figure S4. Determination of the pHPZC of AO-APEI – pH-drift method (Sorbent dosage, SD: 2 g L^{-1}; Time: 48 h; Background salt: 0.1 M NaCl).

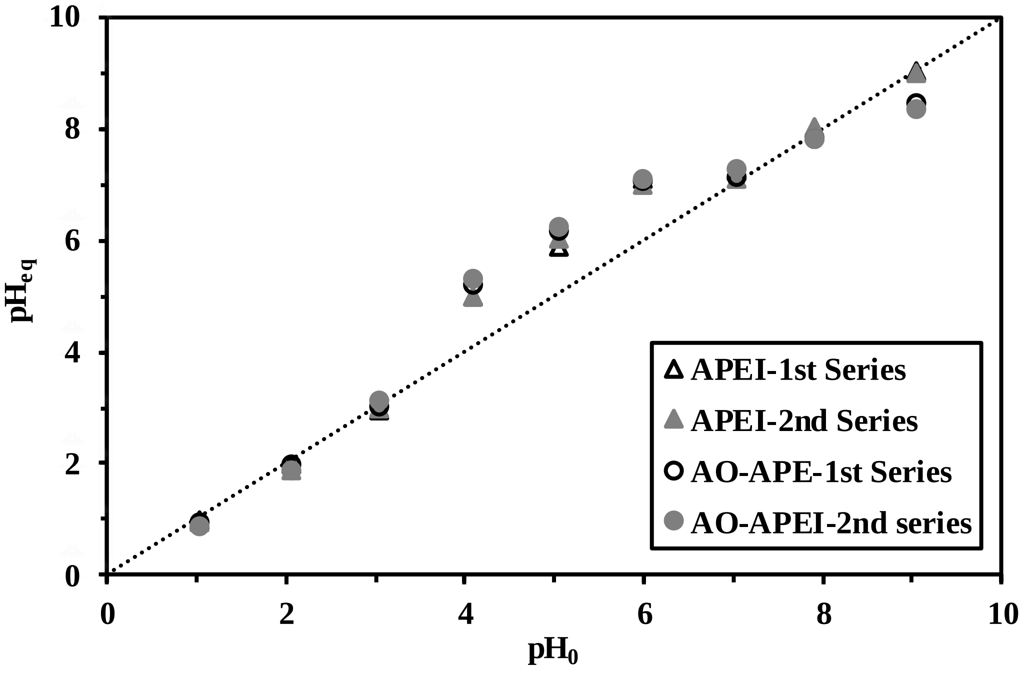

Figure S5. pH variation during Sr(II) sorption using APEI and AO-APEI sorbents – (Duplicate experiments; Sorbent dosage, SD: 0.375 g L^{-1}; C_0 : 29.7 mg Sr L^{-1},; room temperature: 25 ± 1 °C; agitation speed: 140 rpm; contact time: 48 h).

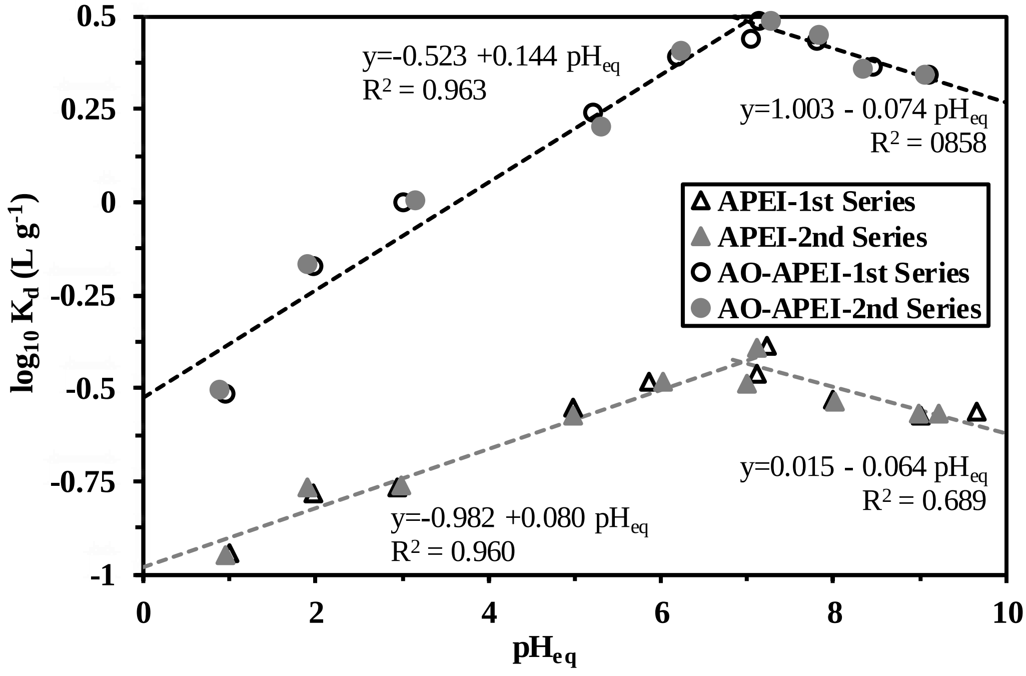

Figure S6. Effect of pH on the distribution coefficient (log10 units) for Sr(II) onto APEI and AO-APEI sorbents – (Duplicate experiments; Sorbent dosage, SD: 0.375 g L^{-1}; C_0 : 29.7 mg Sr L^{-1}; room temperature: 25 ± 1 °C; agitation speed: 140 rpm; contact time: 48 h).

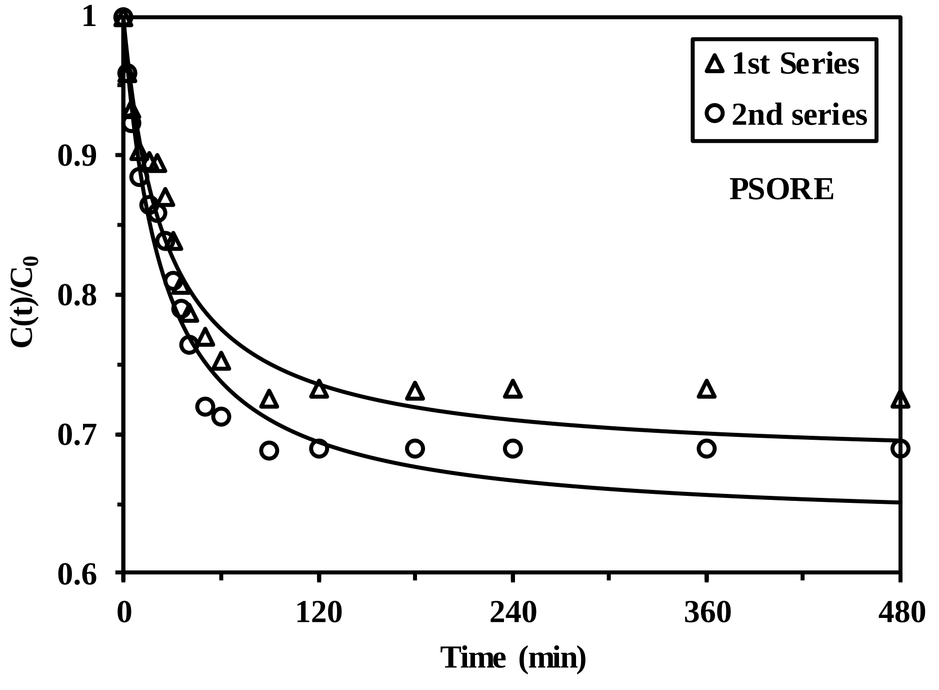

Figure S7. Uptake kinetics for Sr(II) removal using AO-APEI sorbent – Modeling of kinetic profiles with the PSORE model (pH_0: 6.0; pH_{eq}: 7.1; Sorbent dosage, SD: 0.2 g L^{-1}; C_0 : 55.5 and 50.0 mg Sr L^{-1} for 1st and 2nd series, respectively; Temperature: 25 ± 01 °C; agitation speed: 140 rpm).

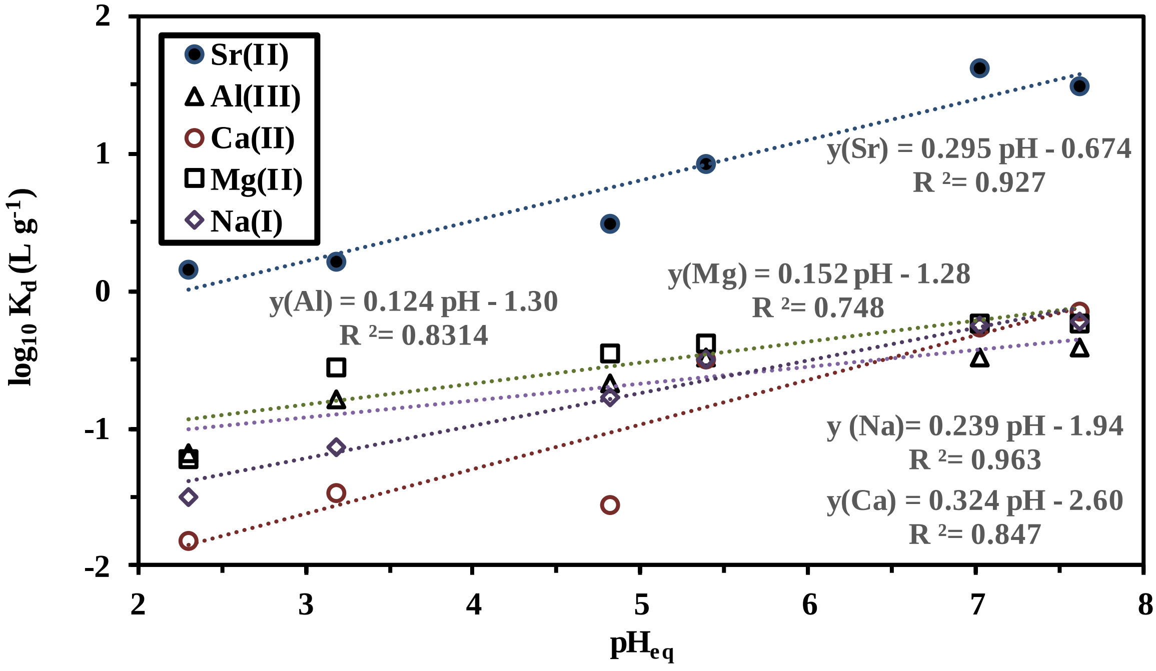

Figure S8. Effect of pH on distribution coefficients (log_{10} units) for Sr(II), Al(III), Ca(II) Mg(II) and Na(I) recovery using AO-APEI from equimolar multi-component solutions (1.8 mmol L^{-1}; SD: 125 mg L^{-1}; Temperature: 25 ± 1 °C; agitation speed: 140 rpm; contact time: 48 h).

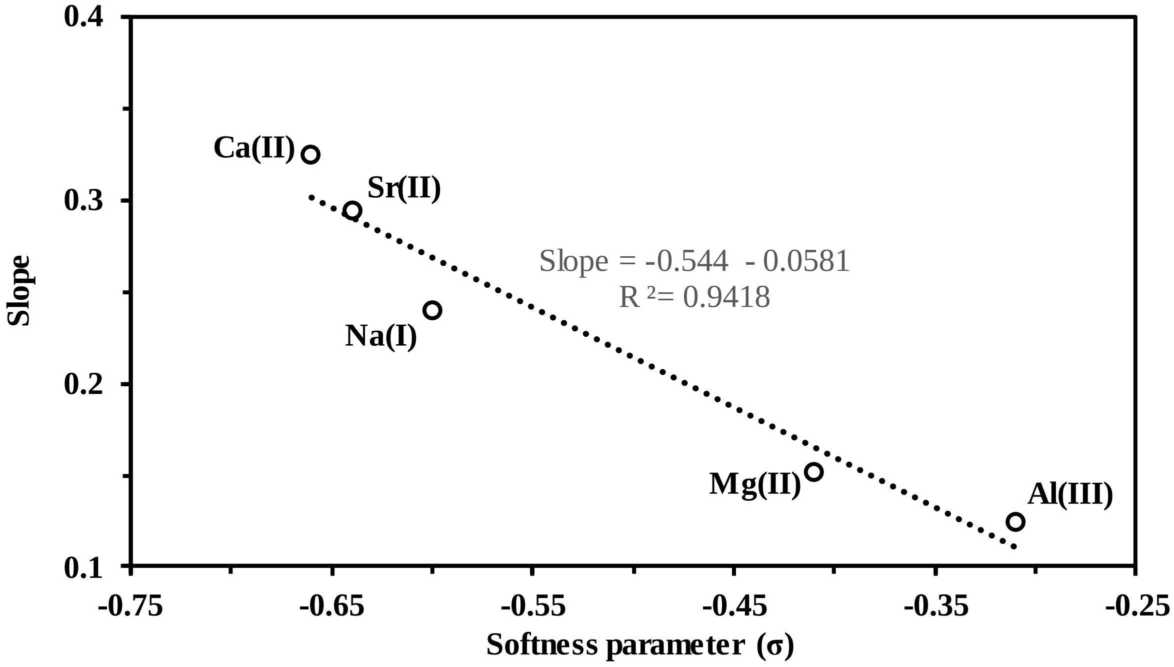

Figure S9. Correlation between the slope of the plots $\log_{10} K_d$ *vs.* pH and the softness parameter of selected metal ions (data collected from Figure S8 and Marcus).[18]

Na, Mg and Al are octahedron while Ca and Sr are square antiprism.

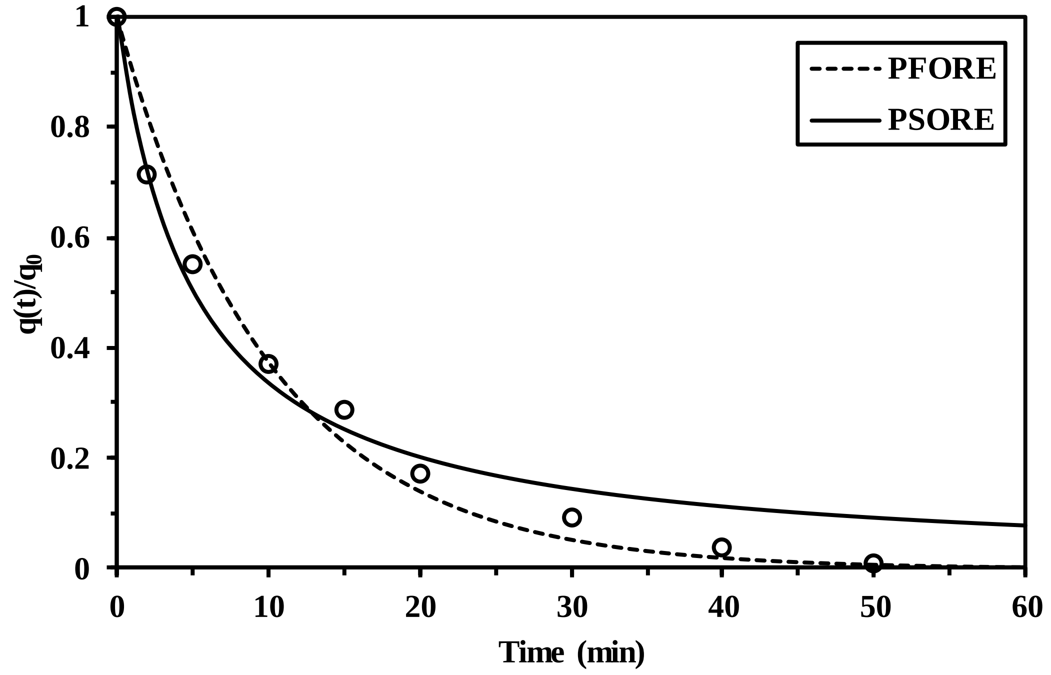

Figure S10. Desorption kinetics for Sr(II) elution from AO-APEI sorbent using 0.5 M $CaCl_2$/0.2 M HCl solution – Modeling of kinetic profiles using the PFORE and PSORE adapted to desorption (SD: 1 g L^{-1}; q_0: 76 mg Sr g^{-1} = 0.867 mmol Sr g^{-1}; Temperature: 21 ± 1 °C; agitation speed: 140 rpm).

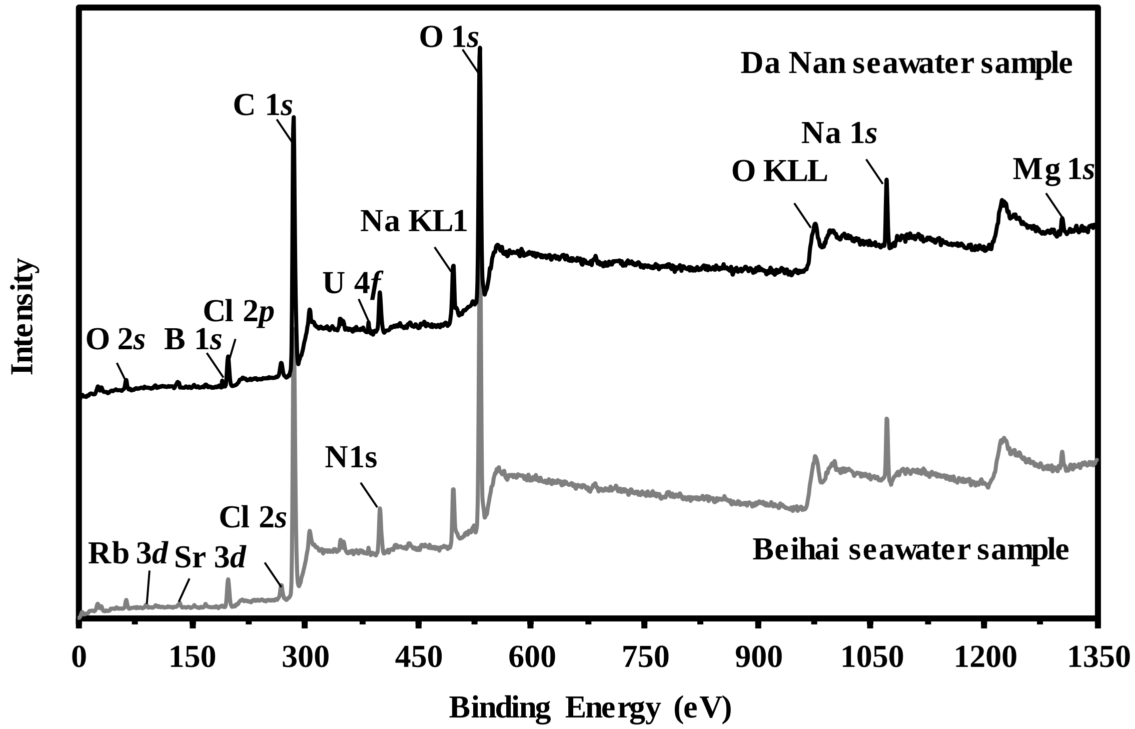

Figure S11. XPS (survey) spectra of AO-APEI beads after being in contact with seawater samples (Beihai, China and Da Nan, Vietnam).

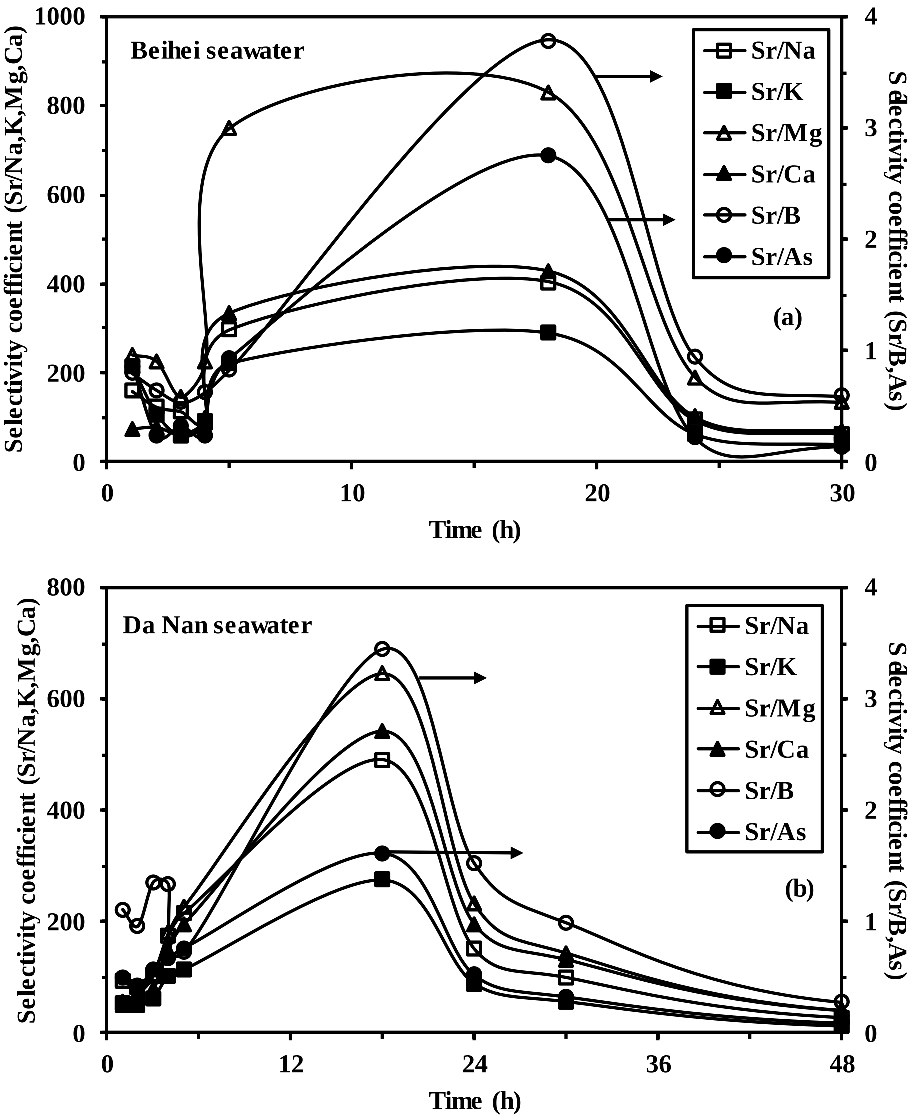

Figure S12. Selectivity for Sr(II) removal from 2 samples of seawater (SD: 0.2 g L^{-1}; pH_0: 7.98 (Da Nan seawater sample), 7.63 (Biehei seawater sample)).

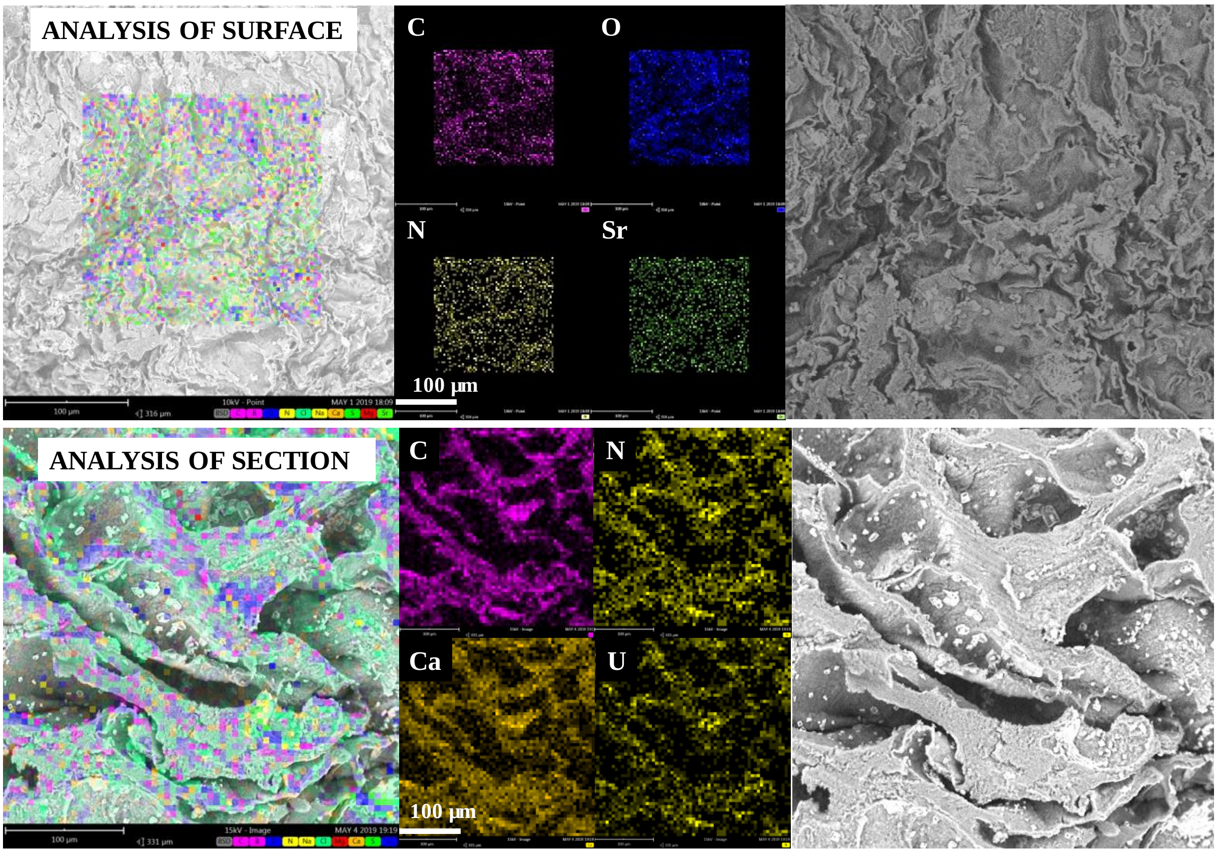

Figure S13. Example of EDX analysis (element cartography) of AO-APEI beads after contact with seawater (analysis of surface and section).

References

1. Hamza, M. F.; Aly, M. M.; Abdel-Rahman, A. A. H.; Ramadan, S.; Raslan, H.; Wang, S.; Vincent, T.; Guibal, E., Functionalization of magnetic chitosan particles for the sorption of U(VI), Cu(II) and Zn(II)—Hydrazide derivative of glycine-grafted chitosan. *Materials* **2017,** 10, (5), 539-560.
2. Hamza, M. F.; Abdel-Rahman, A. A. H., Extraction studies of some hazardous metal ions using magnetic peptide resins. *J. Dispersion Sci. Technol.* **2015,** 36, (3), 411-422.
3. Coates, J., Interpretation of Infrared Spectra, A Practical Approach. In *Encyclopedia of Analytical Chemistry*, Meyers, R. A., Ed. John Wiley & Sons Ltd: Chichester, U.K., 2000; pp 10815-10837.
4. Mohammadi, N.; Ganesan, A.; Chantler, C. T.; Wang, F., Differentiation of ferrocene D-5d and D-5h conformers using IR spectroscopy. *J. Organomet. Chem.* **2012,** 713, 51-59.
5. Colthup, N. B.; Daly, L. H.; Wiberley, S. E., *Introduction to Infrared and Raman Spectroscopy*. 3rd. ed. ed.; Academic Press, Inc.: San Diego, CA (USA), 1990; p 560.
6. Hu, X. J.; Wang, J. S.; Liu, Y. G.; Li, X.; Zeng, G. M.; Bao, Z. L.; Zeng, X. X.; Chen, A. W.; Long, F., Adsorption of chromium (VI) by ethylenediamine-modified cross-linked magnetic chitosan resin: Isotherms, kinetics and thermodynamics. *J. Hazard. Mater.* **2011,** 185, (1), 306-314.
7. Hosoba, M.; Oshita, K.; Katarina, R. K.; Takayanagi, T.; Oshima, M.; Motomizu, S., Synthesis of novel chitosan resin possessing histidine moiety and its application to the determination of

trace silver by ICP-AES coupled with triplet automated-pretreatment system. *Anal. Chim. Acta* **2009,** 639, (1-2), 51-56.

8. Oshita, K.; Takayanagi, T.; Oshima, M.; Motomizu, S., Adsorption behavior of cationic and anionic species on chitosan resins possessing amino acid moieties. *Anal. Sci.* **2007,** 23, (12), 1431-1434.
9. Cao, P. G.; Yao, J. L.; Bin, R.; Gu, R. N.; Tian, Z. Q., Surface-enhanced Raman scattering spectra of thiourea adsorbed at an iron electrode in $NaClO_4$ solution. *J. Phys. Chem. B* **2002,** 106, (39), 10150-10156.
10. Namdeo, M.; Bajpai, S. K., Chitosan-magnetite nanocomposites (CMNs) as magnetic carrier particles for removal of Fe(III) from aqueous solutions. *Colloids Surf., A* **2008,** 320, (1-3), 161-168.
11. Zhang, X.; Jiao, C.; Wang, J.; Liu, Q.; Li, R.; Yang, P.; Zhang, M., Removal of uranium(VI) from aqueous solutions by magnetic Schiff base: Kinetic and thermodynamic investigation. *Chem. Eng. J.* **2012,** 198, 412-419.
12. Zhang, T.; Tu, Z.; Lu, G.; Duan, X.; Yi, X.; Guo, C.; Dang, Z., Removal of heavy metals from acid mine drainage using chicken eggshells in column mode. *J. Environ. Manage.* **2017,** 188, 1-8.
13. Lazaridis, N. K.; Pandi, T. A.; Matis, K. A., Chromium(VI) removal from aqueous solutions by Mg-Al-CO_3 hydrotalcite: Sorption-desorption kinetic and equilibrium studies. *Ind. Eng. Chem. Res.* **2004,** 43, (9), 2209-2215.
14. Ho, Y. S.; McKay, G., Pseudo-second order model for sorption processes. *Process Biochem.* **1999,** 34, (5), 451-465.
15. Crank, J., *The Mathematics of Diffusion*. 2nd. ed.; Oxford University Press: Oxford, U.K., 1975; p 414.
16. Tien, C., *Adsorption Calculations and Modeling*. Butterworth-Heinemann: Newton, MA, 1994; p 243.
17. Foo, K. Y.; Hameed, B. H., Insights into the modeling of adsorption isotherm systems. *Chem. Eng. J.* **2010,** 156, (1), 2-10.
18. Marcus, Y., *Ion Properties*. Marcel Dekker, Inc.: New York, NY, 1997; p 259.
